# Preclinical efficacy of CBR-5884 against epithelial ovarian cancer cells by targeting the serine synthesis pathway

**DOI:** 10.1007/s12672-024-01013-0

**Published:** 2024-05-11

**Authors:** Kunxiang Gong, Yinger Huang, Yanqin Zheng, Yinfu Zhu, Wenbo Hao, Kun Shi

**Affiliations:** 1 Institute of Reproductive Health and Perinatology, Guangzhou Women and Children’s Medical Center, Guangzhou Medical University, Guangzhou, 510623 China; 2 Department of Gynecology and Obstetrics, Guangzhou Women and Children’s Medical Center, Guangzhou Medical University, Guangzhou, 510623 Guangdong China; 3 School of Traditional Chinese Medicine, Southern Medical University, Guangzhou, 510515 Guangdong China; 4 Institute of Antibody Engineering, School of Laboratory Medicine and Biotechnology, Southern Medical University, Guangzhou, 510515 Guangdong China

**Keywords:** Epithelial ovarian cancer, CBR-5884, Serine biosynthesis, PHGDH, Patient-derived organoid model

## Abstract

Reprogramming of the serine synthesis pathway (SSP) is intricately linked to the progression of epithelial ovarian cancer (EOC). CBR-5884, a selective small-molecule inhibitor targeting phosphoglycerate dehydrogenase (PHGDH), effectively impedes the de novo synthesis of serine within cancer cells. This study aimed to evaluate the inhibitory effect of CBR-5884 on EOC cells and delineate its specific mechanism, thereby proposing a novel therapeutic approach for treating EOC. The suppression of serine biosynthesis after CBR-5884 treatment was evaluated using RNA sequencing and a serine assay kit, and the results showed that CBR-5884 effectively downregulated serine biosynthesis in EOC cells, particularly those expressing high levels of PHGDH. In vitro studies revealed that CBR-5884 demonstrated significant antitumor effects and suppressed migration and invasion of EOC cells through down-regulation of the integrin subunit beta 4 (ITGB4)/extracellular signal-regulated kinase (ERK)/epithelial–mesenchymal transition signal axis. Additionally, CBR-5884 mitigated the stemness of EOC cells and heightened their sensitivity to chemotherapy. Moreover, in vivo studies revealed that CBR-5884 significantly delayed tumor growth, with histological analysis indicating the safety profile of CBR-5884. Finally, the patient-derived organoid (PDO) models were utilized to explore the preclinical efficacy of CBR-5884 against EOC cells, and the results unveiled that CBR-5884 impeded proliferation and downregulated the expression of ITGB4 in EOC PDO models. Our findings supports the anticancer properties of CBR-5884 in EOC cells exhibiting high PHGDH expression, manifesting through the suppression of proliferation, migration, and invasion, while enhancing chemotherapy sensitivity, suggesting that CBR-5884 holds promise as an efficacious strategy for the treatment of EOC.

**Supplementary Information:**

The online version contains supplementary material available at 10.1007/s12672-024-01013-0.

## Introduction

Ovarian cancer (OC) is one of the most prevalent malignant tumors affecting the female reproductive system worldwide [1]. Among the various histological types of OC, epithelial OC (EOC) emerges as the most frequent, constituting more than 90% of all cases [2]. Presently, patients with EOC witness limited advantages from conventional first-line chemotherapy, contributing to persistently high mortality rates [3, 4]. The substantial heterogeneity inherent in OC poses a significant challenge [5]. Therefore, the development of targeted therapeutics is critical for the treatment of patients with OC.

Metabolic reprogramming is a process wherein cancer cells dynamically adapt metabolically to environmental changes, striving to meet the requisite supply for their proliferation [6, 7]. Serine, a fundamental donor of one-carbon units, undergoes de novo synthesis from glucose through the serine synthesis pathway (SSP), serving as a pivotal precursor for the synthesis of nucleotides and lipids [8]. In cancer cells, the SSP is upregulated, thereby crucially contributing to tumor progression and stemness formation [9–11], notably observed in EOC [12]. The heightened activity of SSP intensifies one-carbon unit metabolism, consequently leading to purine metabolic abnormalities closely intertwined with the occurrence and development of EOC [13]. Furthermore, abnormalities in SSP within EOC cells amplify the glycine content, significantly expediting tumor cell proliferation [14]. Ergo, targeting the overactive serine metabolism emerges as a promising strategy for EOC therapy.

Phosphoglycerate dehydrogenase (PHGDH) is the first rate-limiting enzyme of the SSP, catalyzing the conversion of 3-phosphoglycerate into 3-phosphohydroxypyruvate. Overexpression or amplification of PHGDH has been observed in various tumor tissues [15, 16]. Silencing the upregulated PHGDH, which blocks the overactive SSP, has shown a clear antitumor effect [17]. Recently, the role and function of PHGDH in tumor cells have been widely explored. For instance, heightened PHGDH overexpression increases NADPH levels, maintains mitochondrial redox homeostasis, reduces apoptosis, and fosters metastasis in breast cancer cells [18]. Targeting PHGDH-mediated serine synthesis can attenuate brain metastasis in triple-negative breast cancer and enhance overall survival in mice [19]. Moreover, elevated mRNA expression of PHGDH promotes platinum resistance in OC cells [20]. Therefore, utilizing small-molecule inhibitors against PHGDH to inhibit SSP holds promise as a therapeutic strategy for EOC with overexpressed PHGDH. Although a few PHGDH inhibitors have been discovered to date, their development remains limited. CBR-5884, a selective small-molecule inhibitor of PHGDH that impedes de novo serine synthesis, has been identified by screening 800,000 drug-like compounds. CBR-5884 exhibits selective toxicity against cancer cell lines with high activity of serine biosynthesis [21]. CBR-5884 effectively reduces serine content in glioblastoma cells, leading to inhibition of cell proliferation [22]. Furthermore, CBR-5884 significantly overcomes platinum resistance in gastric cancer cells [23]. However, it remains unclear whether CBR-5884 can target the reprogramming of amino acid metabolism in PHGDH overexpressed EOC cells and whether suppressing SSP induced by CBR-5884 could hinder EOC cell development or overcome chemotherapy resistance.

The present study demonstrated that CBR-5884 inhibits overactive serine metabolism and one-carbon unit metabolism in EOC cells, displaying significant antitumor effects both in vitro and in vivo as well as in organoid models. Moreover, CBR-5884 improves EOC cell chemosensitivity by impairing the stemness of EOC cells. Collectively, our study provides an efficacious strategy for EOC treatment.

## Materials and methods

### Cell culture and materials

The SKOV3 (human EOC) cells and MDA-MB-231 (human breast cancer) cells were procured from Procell Life Science & Technology Co., Ltd. (Wuhan, China). The IOSE-80 (normal human ovarian surface epithelial) cells and ID8 (murine EOC) cells were obtained from iCell Bioscience Inc. (Shanghai, China). All cells utilized in this study underwent periodic *Mycoplasma* testing, and their passage number was maintained below 20. Cultivation of MDA-MB-231, IOSE-80, SKOV3, and ID8 cells was performed in Dulbecco’s modified Eagle medium (DMEM; Gibco, USA) supplemented with 10% fetal bovine serum (FBS) (Gibco, USA). All cells were cultured at 37 °C with 5% CO_2_. A selective PHGDH inhibitor (CBR-5884, Cat. No. HY-100012) and carboplatin (CBP, Cat. No. HY-17393) were procured from MedChemExpress (Shanghai, China). Minimum Essential Medium (MEM) (Gibco, USA) was employed to determine serine concentration.

### Transcriptome sequencing and bioinformatics analysis

Total RNA was extracted from dimethyl sulfoxide—(DMSO; control) and CBR-5884 (50 μM)—treated SKOV3 cells, and RNA extraction was carried performed using TriZol reagent (TaKaRa, Japan). Annoroad (Beijing, China) conducted RNA sequencing. Three biological replicates were used for each group. Gene set enrichment analysis (GSEA) [24] was performed for enrichment analysis, with statistical significance defined as P values of < 0.05 and false discovery rate < 0.25. PHGDH mRNA expression data of patients with OC identified from The Cancer Genome Atlas database were obtained from UCSC Xena [25], while data from GSE14407 were acquired using the NCBI tool GEO2R (https://www.ncbi.nlm.nih.gov/geo/geo2r/). Kaplan–Meier Plotter provided the overall survival analysis of PHGDH in GSE26193 for patients with OC [26].

### Serine concentration detection

Briefly, SKOV3 and ID8 cells were seeded into 6-well plates at a density of 5 × 10^5^ cells per well in serum-free MEM and allowed to adhere overnight. The next day, cells were treated with either DMSO control or CBR-5884 (30 μM and 50 μM). After 24 h of treatment, cells were collected, and the cellular serine levels were quantified using a DL-serine assay kit (Cat. No. ab241027; Abcam, MA, USA) following the manufacturer’s instructions.

### Cell viability and 50% inhibiting concentration (IC_50_)

In each well of a 96-well plate, 4 × 10^3^ SKOV3, ID8, and MDA-MB-231 cells were seeded using 100 µL of DMEM supplemented with 10% FBS and allowed to adhere overnight. Subsequently, cells were exposed to test compounds for the specified duration, and cell viability was assessed using the Cell Counting Kit-8 (CCK-8) kit (Selleck Chemicals, USA). After 2-h incubation at 37 °C, absorbance was measured at 450 nm using a microplate reader (iMark™; Bio-Rad Laboratories, Inc.). The results were presented as background-subtracted relative light units normalized to a DMSO-treated control. IC_50_ curves for CBR-5884 were determined by analyzing the cell viability results normalized against untreated controls, and the IC_50_ values was analyzed using GraphPad PRISM 6.0 software. All experiments were conducted in triplicate.

### Colony formation assay

For the assessment of the CBR-5884 effect on SKOV3 and ID8 cells, 5000 cells per well were cultured in a 6-well plate with varying concentrations of CBR-5884 (0, 30, and 60 μM) for 7 days. Following this, the cells were fixed in 4% paraformaldehyde for 10 min and stained with 1% Giemsa for 10 min. Subsequent counting of cell colonies was performed using ImageJ software. This experiment was repeated independently three times.

### Flow cytometry

Flow cytometry experiments were employed to evaluate cell cycle arrest and apoptosis induced by CBR-5884. SKOV3 and ID8 cells were treated with either DMSO or CBR-5884 (50 μM) for 24 or 48 h. For cell cycle analysis, the collected cells were washed twice with phosphate-buffered saline (PBS) subsequently processed using the Cell Cycle Staining Kit (Multi Science, China) according to the manufacturer’s protocol. For apoptosis analysis, cells were washed twice with PBS subsequently stained using the Annexin V-FITC Apoptosis Detection Kit and the Annexin V-APC Apoptosis Detection Kit (MultiScience, China) according to the manufacturer’s instructions. Cells were sorted using a fluorescence-activated cell sorter (Guava easyCyte 8, USA). Cell cycle distributions (S, G1, and G2/M phases) were determined using ModFit LT 3.1 software (Verity Software House, USA), while FlowJo vX 0.7 software (Tree Star Inc., USA) was used to assess apoptosis ratios. To detect CD44-positive cells, a FITC anti-mouse CD44 (IM7; 1:200 dilution, Cat. No. FITC-65117; Proteintech, USA) antibody was used. In brief, SKOV3 and ID8 cells were inoculated in a 6-well plate (2 × 10^4^ cells per well) with or without CBR-5884 (30 μM) for 48 h. Then the collected cells were washed with PBS and were subsequently stained with FITC antimouse CD44 for 5 min. CD44-positive cells were analyzed using FlowJo vX 0.7 software. Each flow cytometry experiment was performed in at least three replicates.

### Wound healing assay

In 6-well plates, SKOV3 and ID8 cells were seeded at confluent densities and incubated in a serum-free medium for 24 h. Using a 10-μL pipette, scratched wounds were created, and cells were washed by PBS and incubated in fresh serum-free medium with DMSO control or CBR-5884 (30 μM) for 48 h. Wound closure was observed at 0 and 48 h, and images were captured to evaluate cell migration rates. The wound-healing rate was quantified by calculating the proportion of the blank region between wound edges at 0 and 48 h. The wound-healing rate represented the proportion of the lost wound area within 48 h relative to the initial area at 0 h.

### Transwell assay

For the Transwell assay, each insert (8 μm pore size, Falcon) was initially pre-coated with Matrigel (Cat. No. CB-40230; Corning, USA) overnight. The upper insert contained 2 × 10^4^ cells, pre-treated with either DMSO control or 50 μM CBR-5884 for 24 h in 100 μL of serum-free medium. The lower chamber was filled with 200 μL of complete medium as the chemoattractant. Cells were allowed to migrate for 48 h, followed by fixation with a 4% formaldehyde solution and staining with hematoxylin. Unmigrated cells on the apical side of the membrane were removed using wet cotton swabs. Subsequently, the migrated cells were captured and counted under a microscope. For each Transwell insert, we counted the total number of invasive cells in 5 random fields under a 100× microscope. The ratios of invasive cells in CBR-5884 treatment group to those cells in the control group were calculated.

### Tumorsphere assay

Briefly, 5 × 10^3^ SKOV3 and ID8 cells were plated as a single-cell suspension on the 6-well ultra-low attachment plates (Corning, USA) in DMEM/F-12 medium supplemented with 20 ng/mL EGF, 10 μg/mL insulin, 0.5 μg/mL hydrocortisone, and B27 (1:50 dilution, Cat. No. 17504044; Gibco, USA). After 24 h, DMSO control or CBR-5884 (50 μM) was added to the plates. Tumorspheres with a diameter > 100 μm were counted, and images were captured after 5 days of treatment. These experiments were conducted three times.

### Quantitative real-time polymerase chain reaction

RNA was isolated from cells using Trizol reagent (TaKaRa, Japan), and cDNA was generated by reverse transcription using a Takara PrimeScript RT Reagent Kit (TaKaRa, Japan), following the manufacturer’s protocol. The resulting cDNA was then amplified using the SYBR Premix Ex-Taq II Kit (TaKaRa, Japan) using a Biosystems 7500 quantitative real-time polymerase chain reaction (PCR) system. All expression data were normalized to GAPDH-encoding transcript levels. Quantitative real-time PCR assay was performed with specific gene primers. Real-time PCR was performed with the following primers: ALDHA1A-fw 5′-GCACGCCAGACTTACCTGTC-3′, ALDH1A1-rev 5′-CCTCCTCAGTTGCAGGATTAAAG-3′; NANOG-fw 5′-CAATGGTGTGACGCAGAAGG-3′, NANOG-rev 5′-GAAGGTTCCCAGTCGGGTTC-3′; GAPDH-fw 5′-TCTGACTTCAACAGCGACAC-3′, GAPDH-rev 5′-CGTTGTCATACCAGGAAATGAG-3′; SOX2-fw 5′-ATGGGTTCGGTGGTCAAGT-3′, SOX2-rev 5′-ATGTGTGAGAGGGGCAG-3′; EPCAM-fw 5′-CTGGCCGTAAACTGCTTTGT-3′, EPCAM-rev 5′-AGCCCATCATTGTTCTGGAG-3′; POU5F1-fw 5′-TCAGCCAAACGACCATCTGC-3′, POU5F1-rev 5′-GGTTTCTGCTTTGCATATCTCCT-3′.

### Western blotting

The cell lysates were extracted using radioimmunoprecipitation assay lysis buffer (KeyGEN BioTECH, China) containing 1% 100 mM phenylmethanesulfonyl fluoride (KeyGEN BioTECH, China). Total protein was determined utilizing a BCA Protein Assay Kit (KeyGEN BioTECH, China). The isolated proteins from cell lysates were separated on 8–15% sodium dodecyl sulfate–polyacrylamide gel electrophoresis, depending on the specific experiment, and then transferred onto a polyvinylidene difluoride membrane (Millipore, USA) using an electro-transferred machine. Following this, the membranes were blocked with 5% skimmed milk for 1.5 h According to the molecular weight of the target protein, the membranes were cut into several strips at appropriate positions and incubated with primary antibodies against various targets: PHGDH (1:2000 dilution, Cat. No. 14719-1-AP; Proteintech, USA), GAPDH (1:2000 dilution, Cat. No. 5174; Cell Signaling Technology, USA), caspase-3 (1:1000 dilution, Cat. No. 9662; Cell Signaling Technology, USA), cleaved caspase-3 (1:1000 dilution, Cat. No. 9664; Cell Signaling Technology, USA), ITGB4 (1:1000 dilution, Cat. No. 14803; Cell Signaling Technology, USA), phospho-p44/42 MAPK (Erk1/2) (1:2000 dilution, Cat. No. 9102; Cell Signaling Technology, USA), total-p44/42 MAPK (Erk1/2) (1:1000 dilution, Cat. No. 4370; Cell Signaling Technology, USA), ZEB1 (1:1000 dilution, Cat. No. 3396; Cell Signaling Technology, USA), N-cadherin (1:1000 dilution, Cat. No. 13116; Cell Signaling Technology, USA), β-Catenin (1:1000 dilution, Cat. No. 8480; Cell Signaling Technology, USA), and Vimentin (1:1000 dilution, Cat. No. 5741; Cell Signaling Technology, USA). The membranes were incubated with these primary antibodies at 4 °C overnight. Subsequently, the membranes underwent incubation with horseradish peroxidase (HRP)—conjugated secondary antibodies for 1 h at room temperature (anti-rabbit IgG, 1:10,000 dilution, Cat. No. 7074; Cell Signaling Technology). Visualization of protein bands was achieved using chemiluminescence HRP substrate (Millipore, USA). Each western blot image is representative of three repeated results. The normalized densitometry data were determined using ImageJ software. The full scans of original blots were shown in the supplemental material.

### Animal experiments

The study utilized 12 female BALB/c nude mice aged 4–6 weeks, procured from the Experimental Animal Center of Southern Medical University. These mice were housed in autoclaved, ventilated cages and provided with autoclaved water. All mice used were bred in the specific pathogen free laboratory. The mice received a subcutaneous injection of 1 × 10^6^ ID8 cells in the right armpit. When the tumors reached an average volume of 65 mm^3^, the mice were randomly divided into two groups using the random number method: (1) an experimental group, wherein the mice received intragastric administration of CBR-5884 (70 mg/kg, qd; n = 6); and (2) a control group, wherein the mice received intragastric administration of an equal volume of vehicle (corn oil) (n = 6). CBR-5884 treatment lasted for 12 consecutive days, with continuous drug administration. Tumors were resected when the average tumor volume in the control group reached 400 mm^3^. Tumor growth was tracked using caliper measurements, and tumor volume was calculated using the formula: length × width^2^/2. Subsequently, images of mice and tumors were captured, and tumors were weighed. Liver, spleen, kidney, and tumors were subjected to histological analysis after hematoxylin–eosin (H&E) staining. The maximal tumor size/burden permitted by our institutional review board is 10% of body weight and mean tumor diameter = or > 15 mm in adult mice (~ 25 g). The maximal tumor size/burden permitted by our institutional review board was not exceeded.

### Immunohistochemistry, immunofluorescence analysis, and hematoxylin–eosin staining

For immunohistochemistry (IHC) analysis, an IHC kit (Cat. No. IHC001; Bioss, Beijing, China) was employed. Slices were subjected to heating, dewaxing, and rehydration and were then placed in sodium citrate buffer (pH buffer = 6.0) for antigen repair. To inhibit endogenous peroxidase activity, the slides were treated with 3% hydrogen peroxide and sealed with 10% normal sheep serum. After rinsing three times, the slices were incubated overnight with the primary antibody (rabbit anti-Ki-67, 1:500 dilution, Cat. No. 9027; Cell Signaling Technology, USA; rabbit anti-PHGDH, 1:500 dilution, Cat. No. 14719-1-AP; Proteintech, USA; rabbit anti-ITGB4, 1:300 dilution, Cat. No. 14803; Cell Signaling Technology, USA) at 4 °C. Following PBS washes, the slices were treated with a secondary antibody (anti-rabbit IgG 1:2000 dilution, Cat. No. 7074; Cell Signaling Technology, USA) for 30 min at 37 °C. Subsequently, staining with 3BI-3-diaminobenzidine (DAB) was performed, followed by hematoxylin staining, dehydration, sealing, and observation. Three independent pathologists evaluated Ki-67 scores based on the percentage of positive tumor cells. For immunofluorescence assay, the slices were blocked, followed by incubation with the primary antibody (rabbit anti-ITGB4, 1:200 dilution, Cat. No. 14803; Cell Signaling Technology, USA) overnight at 4 °C. Slices were then washed, incubated with secondary antibody (Cy3-conjugated anti-rabbit IgG 1:100 diluted, Cat. No. SA00009-2; ProteinTech, USA), and stained with DAPI (Cat. D-9106, Bioss). H&E staining involved staining sections in hematoxylin for 5 min and eosin for 5 min.

### Patient-derived organoid model construction and culture

Fresh tumor tissues from patients with EOC were immersed in advanced DMEM/F12 (Cat. No. 12634028; Thermo Fisher Scientific) with 1% penicillin–streptomycin (PS). Necrotic tissues were removed, and the remaining tissue was cut into pieces of approximately 1–2 mm^3^ volume. After washing twice with PBS containing 1% PS, the tissue fragments were digested at 37 °C for 1 h using Advanced DMEM/F12 solution with type IV Collagenase (Cat. No. 17104019; Thermo Fisher Scientific). The resulting solution was filtered using a 100-μm filter (Cat. No. 352360; Corning, New York, USA). The fragments were then centrifuged at 300 × g for 5 min, erythrocytes lysed, and the precipitated cells resuspended in Advanced DMEM/F12 mixed with growth factor-reduced Matrigel (Cat. No. CB-40230; Corning, USA). The cell suspension was adjusted to a final concentration of 10,000–20,000 cells/50 μL. Patient-derived organoids (PDOs) were transferred to 24-well plates upon Matrigel solidification, and 500 μL of general culture medium was added. PDOs were cultured in a 37 °C cell incubator with 5% CO_2_, and the culture medium was refreshed every 2–3 days. Cell viability of PDOs was assessed using the Cell Counting-Lite 3D Luminescent Cell Viability Assay (Cat. No. DD1102-01; Vazyme, Nanjing, China).

### Statistical analysis

Data were presented as mean ± standard deviation. Statistical analyses were conducted using GraphPad PRISM 6.0 software. Significant differences between two groups were determined using Student’s t-test (two-tailed). The two-way analysis of variance was performed to evaluate the therapeutic effect of CBR-5884 combined with CBP, and to assess differences in time curves and tumor volume growth curves. P values < 0.05 (*P < 0.05, **P < 0.01, ***P < 0.001) were considered significant; otherwise, they were deemed non-significant (ns).

## Results

### CBR-5884 exerted on-target inhibition on serine metabolism in EOC cells

**Fig. 1:**
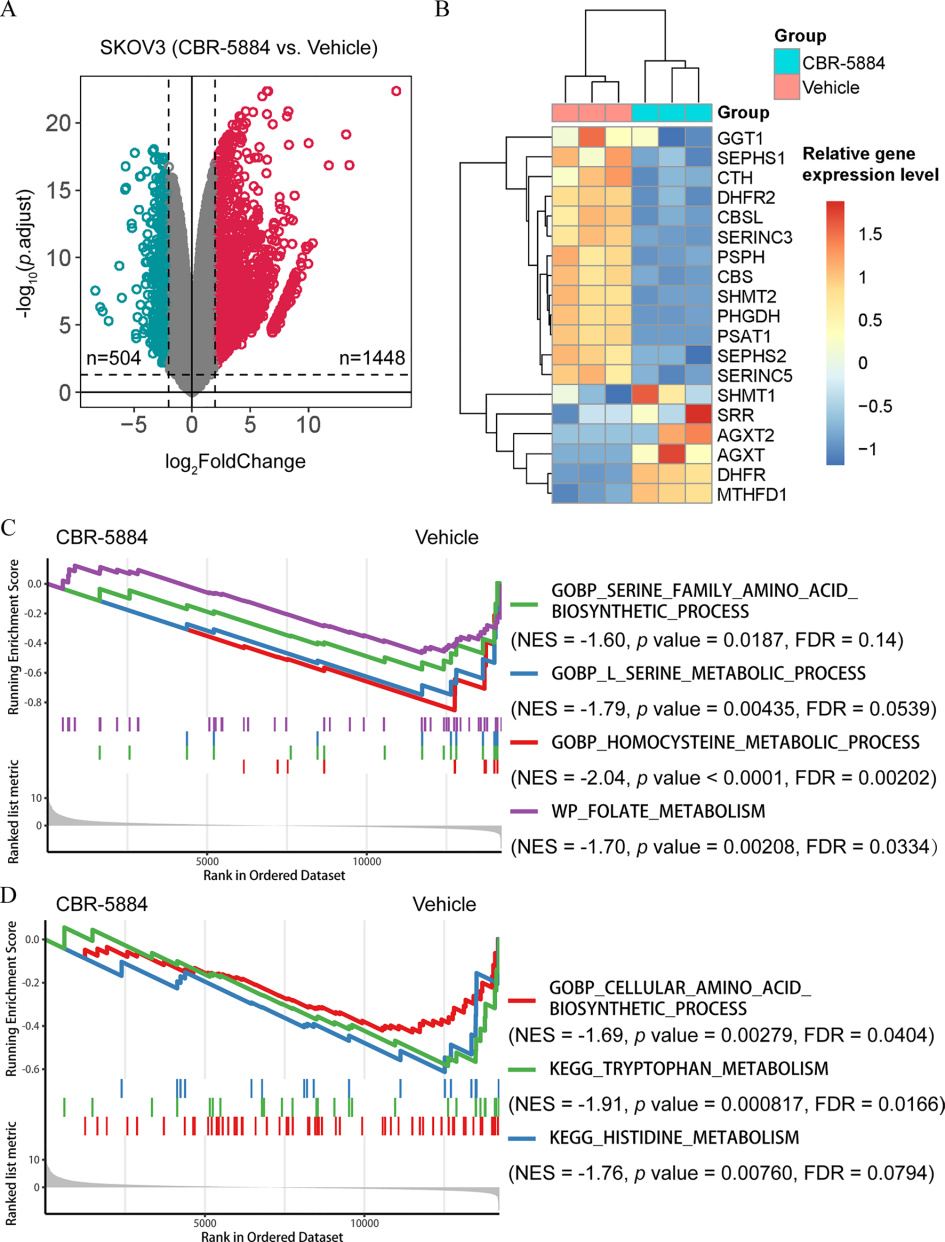
CBR-5884 targeted active serine metabolism in EOC cells. **A** SKOV3 cells were treated with CBR-5884 (50 μM) or dimethyl sulfoxide (control) for 24 h, and a volcano plot was constructed to compare the different genes between the two groups. **B** A heatmap was constructed to compare serine synthesis–related genes after CBR-5884 (50 μM) treatment for 24 h in SKOV3 cells. **C**, **D** Gene set enrichment analysis was performed between CBR-5884-treated SKOV3 cells and control SKOV3 cells

PHGDH has been identified as a potential antitumor therapeutic target, showing increased expression in primary ovarian tumors (Fig. S1A). Elevated PHGDH mRNA expression levels are indicative of poorer overall survival in patients with OC (Fig. S1B). Recently, CBR-5884 was recognized as a selective small-molecule inhibitor of PHGDH. To assess the effect of CBR-5884 on EOC cells, SKOV3 cells were treated with CBR-5884 (50 μM) or a DMSO control for 24 h. Subsequently, RNA sequencing was conducted, followed by analysis to compare the transcriptomic variances between the two groups (Fig. S1C). Differential gene expression analysis, using a screening threshold of |log_2_FoldChange| > 2 and p.adjust < 0.05, revealed that 1448 genes were upregulated and 504 genes were downregulated following CBR-5884 treatment (Fig. 1A). Notably, the mRNA expression of genes associated with serine synthesis was notably reduced within 24 h in SKOV3 cells treated with CBR-5884 (Fig. 1B). Additionally, GSEA was conducted to investigate alterations in pathway activity upon CBR-5884 treatment. Interestingly, the gene sets GOBP_SERINE_FAMILY_AMINO_ACID_BIOSYNTHETIC_PROCESS and GOBP_L_SERINE_METABOLIC_PROCESS were significantly enriched in the control group rather than in the CBR-5884 group, suggesting effective inhibition of cellular serine biosynthesis by the compound. Serine plays a crucial role as a cellular one-carbon unit source. As expected, CBR-5884 in OC cells inhibited processes related to one-carbon metabolism, evident in the GOBP_HOMOCYSTEINE_METABOLIC_PROCESS and WP_FOLATE_METABOLISM (Fig. 1C). Furthermore, the treatment with CBR-5884 seemed to repress the activity of amino acid metabolism, specifically affecting tryptophan and histidine metabolism, possibly due to shared crosstalk or similarities between these metabolic processes (Fig. 1D). In summary, these findings suggest that CBR-5884 inhibits serine biosynthesis and related metabolic pathways crucial for the malignant behavior of cancers, particularly in EOC.

### CBR-5884 inhibited the proliferation of EOC cells

**Fig. 2:**
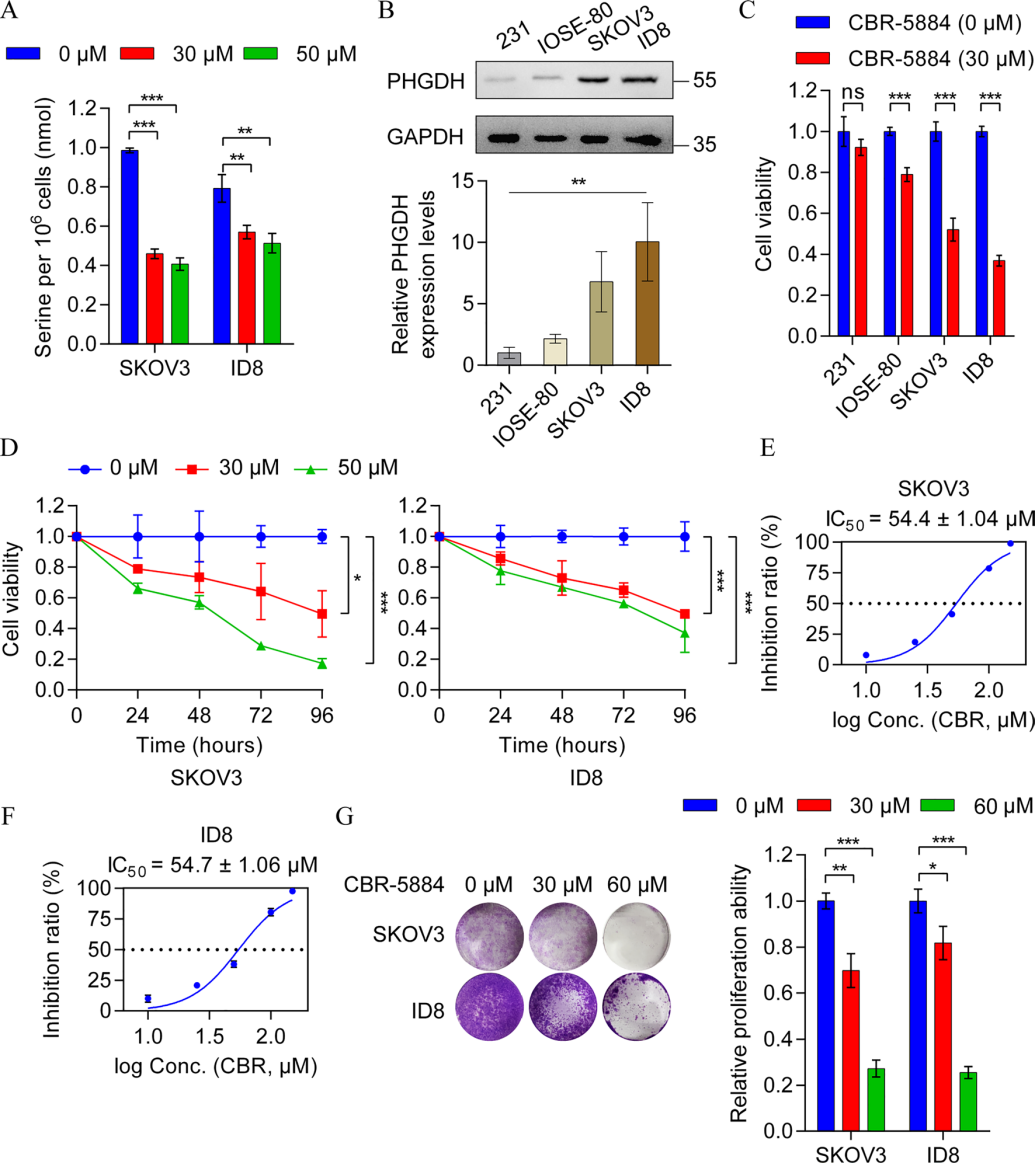
CBR-5884 suppressed the growth of EOC cells. **A** Determination of serine content in SKOV3 and ID8 cells treated with CBR-5884 (0 μM, 30 μM, and 50 μM) for 24 h. **B** Western blot analysis to detect the protein levels of phosphoglycerate dehydrogenase (PHGDH) in MDA-MB-231, IOSE-80, SKOV3, and ID8 cells. Representative images (top panel) and quantification data (bottom panel) are shown. **C** CCK-8 assay was performed to determine the cell viability in MDA-MB-231, IOSE-80, SKOV3, and ID8 cells treated with CBR-5884 (30 μM) for 72 h. **D** SKOV3 and ID8 cells were treated with different concentrations of CBR-5884 (0 μM, 30 μM, and 50 μM) for 4 days, and CCK-8 assay was performed to detect the inhibitory effect of CBR-5884 treatment on cell proliferation. **E**, **F** CCK-8 assay was performed to measure the IC_50_ curves of SKOV3 and ID8 cells treated with different concentrations of CBR-5884 for 72 h. **G** A clone formation test was used to evaluate the inhibitory role of CBR-5884 in the clone formation of ovarian cancer cells. Quantitative data of relative area intensity was compared and calculated. Representative images (left) and quantification data (right) are shown

To evaluate the inhibitory effect of CBR-5884 on serine de novo synthesis, we assessed the total serine content in EOC cell lines SKOV3 and ID8 treated with varying concentrations of CBR-5884 for 24 h. The findings demonstrated a substantial reduction in endogenous serine availability due to CBR-5884 treatment (Fig. 2A). To corroborate the lethal effect of CBR-5884 on cells with heightened serine synthesis propensity, we examined PHGDH protein expression levels in MDA-MB-231 (a breast cancer cell line), IOSE-80 (a normal human ovarian surface epithelial cell line), and the EOC cell lines SKOV3 and ID8. Western blot analysis revealed elevated PHGDH expression in both EOC cells compared to MDA-MB-231 and IOSE-80 (Fig. 2B). Subsequently, all four cell lines were treated with 30 μM CBR-5884 for 72 h. Notably, CBR-5884 exhibited minimal effect on MDA-MB-231 and IOSE-80 cells but significantly impeded the proliferation of high PHGDH-expressing EOC cells (Fig. 2C). Furthermore, CBR-5884 consistently suppressed EOC cell proliferation in a time-dependent manner (Fig. 2D). Subsequent determination of the IC_50_ values of CBR-5884 in SKOV3 and ID8 cells treated with varying concentrations (0–150 μM) for 72 h indicated values of 54.4 μM in SKOV3 cells and 54.7 μM in ID8 cells (Fig. 2E, F). Additionally, the clone formation assay conspicuously illustrated the inhibition of EOC cell growth by CBR-5884 (Fig. 2G and Fig. S2). Overall, these findings suggest that CBR-5884 restrains endogenous serine synthesis, leading to proliferation inhibition in EOC cells expressing high levels of PHGDH.

### CBR-5884 blocked the cell cycle and induced apoptosis of EOC cells

**Fig. 3:**
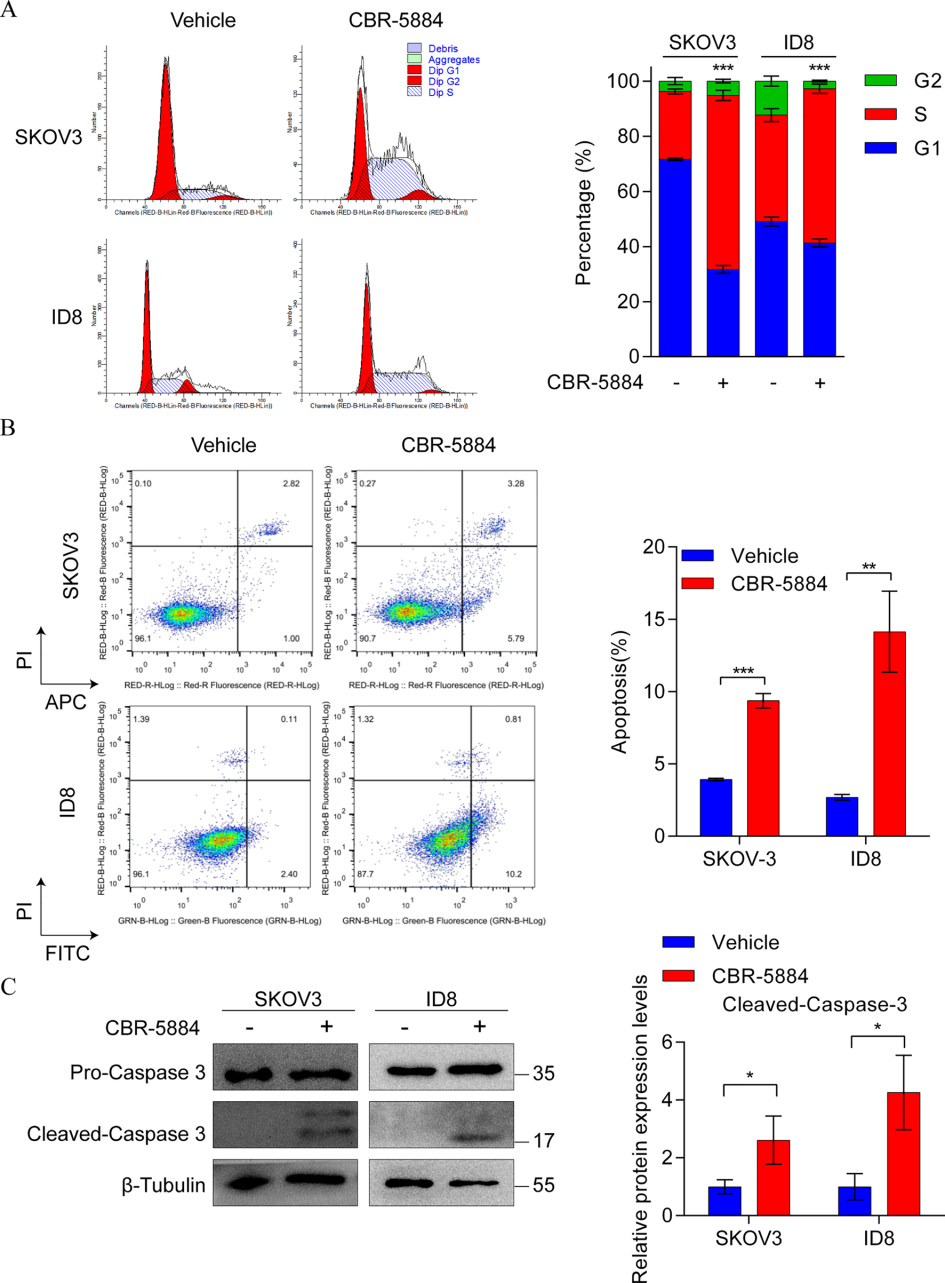
CBR-5884 effectively induced cell cycle arrest and apoptosis in EOC cells. **A** Cell cycle distribution of SKOV3 and ID8 cells incubated without and with CBR-5884 (50 μM) for 24 h was determined by flow cytometry. **B** Apoptosis of SKOV3 and ID8 cells treated with 50 μM CBR-5884 for 48 h was investigated by flow cytometry. **C** Western blot was performed to detect the protein expression levels of cleaved-caspase 3 in SKOV3 and ID8 cells treated with CBR-5884 (50 μM) for 48 h. Representative results (left) and quantification data (right) are shown

Beyond the effect on cell proliferation, we investigated the effect of CBR-5884 on the cell cycle in EOC cells. Treatment with CBR-5884 (50 μM) for 24 h resulted in cell cycle arrest primarily at the S phase in SKOV3 and ID8 cells (Fig. 3A). To assess whether CBR-5884-induced growth arrest of EOC cells was linked to apoptosis induction, we evaluated apoptotic cells via flow cytometry and measured the expression levels of cleaved caspase-3 through western blot analysis. Intriguingly, treatment with CBR-5884 (50 μM) for 48 h notably increased the percentage of Annexin V-positive cells (Fig. 3B) and activated the expression of cleaved-Caspase 3 in both SKOV3 and ID8 cells (Fig. 3C). These collective observations reveal that CBR-5884, a selective PHGDH inhibitor, effectively inhibits the growth of EOC cells by inducing cell cycle arrest and apoptosis, indicating its potential as a treatment option for EOC.

### CBR-5884 suppressed the migration and invasion of EOC cells by downregulating the ITGB4/ERK/EMT axis

**Fig. 4:**
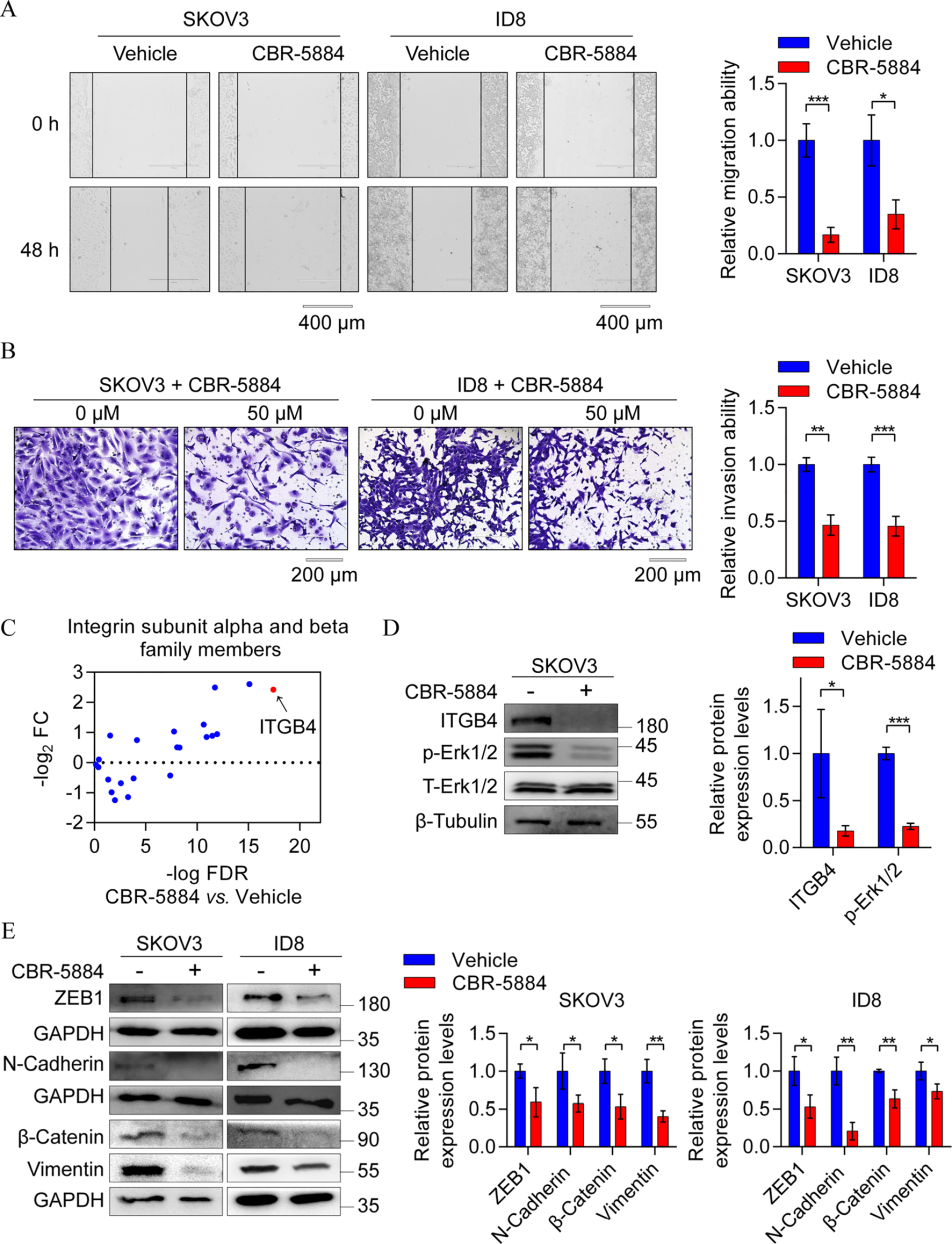
CBR-5884 suppressed the migration and invasion of EOC cells. **A** A wound healing assay was performed to assess the migration ability of SKOV3 and ID8 cells after CBR-5884 (30 μM) treatment for 48 h. **B** SKOV3 and ID8 cells were pretreated with 50 μM CBR-5884 for 24 h, and the Transwell assay was used to detect the cell invasive ability. **C** mRNA expression levels of integrin family members were downregulated by CBR-5884 treatment, and the red dot represents ITGB4. **D** Western blot analysis was performed to verify the downregulated expression of genes related to the ITGB4/ERK pathway after treatment with 50 μM CBR-5884 for 48 h in SKOV3 cells. **E** Western blot assay was performed to detect proteins related to the epithelial–mesenchymal transition in SKOV3 and ID8 cells treated with CBR-5884 (50 μM) for 48 h. Representative images (left) and quantification data (right) are shown

As CBR-5884 demonstrates the ability to impede cell growth and induce apoptosis in EOC cells, this study investigated its potential inhibitory effect on cell migration and invasion. To explore this, SKOV3 and ID8 cells were exposed to CBR-5884 (30 μM) for 48 h, observing a marked decrease in cell migration in both cell lines (Fig. 4A). Furthermore, the invasive ability of tumor cells notably diminished after 24 h of treatment with 50 μM CBR-5884 (Fig. 4B). Notably, our sequencing data revealed significant downregulation of integrin family members’ mRNA expression levels upon exposure to CBR-5884, with integrin subunit beta 4 (ITGB4) exhibiting the most significant downregulation (Fig. 4C). ITGB4, functioning as a membrane receptor, when stimulated extracellularly, interacts with receptor tyrosine kinases, activating the downstream extracellular signal-regulated kinase (ERK) signaling pathway, thus facilitating tumor cell metastasis [27, 28]. Subsequently, SKOV3 cells were treated with CBR-5884 (50 μM) for 48 h, and western blot results validated the remarkable downregulation of the ITGB4/ERK pathway (Fig. 4D). The activation of ERK typically sustains the epithelial–mesenchymal transition (EMT) of EOC cells, promoting their metastasis [29]. Therefore, we analyzed the expression levels of EMT-related proteins, including ZEB1, N-Cadherin, β-Catenin, Vimentin. Western blot results from both SKOV3 and ID8 cells treated with 50 μM CBR-5884 for 48 h demonstrated a significant down-regulation of these proteins (Fig. 4E). This suggests that CBR-5884 impedes the migration and invasion of EOC cells by suppressing the ITGB4/ERK/EMT signaling axis.

### CBR-5884 enhanced chemotherapy sensitivity by impairing EOC cell stemness

**Fig. 5:**
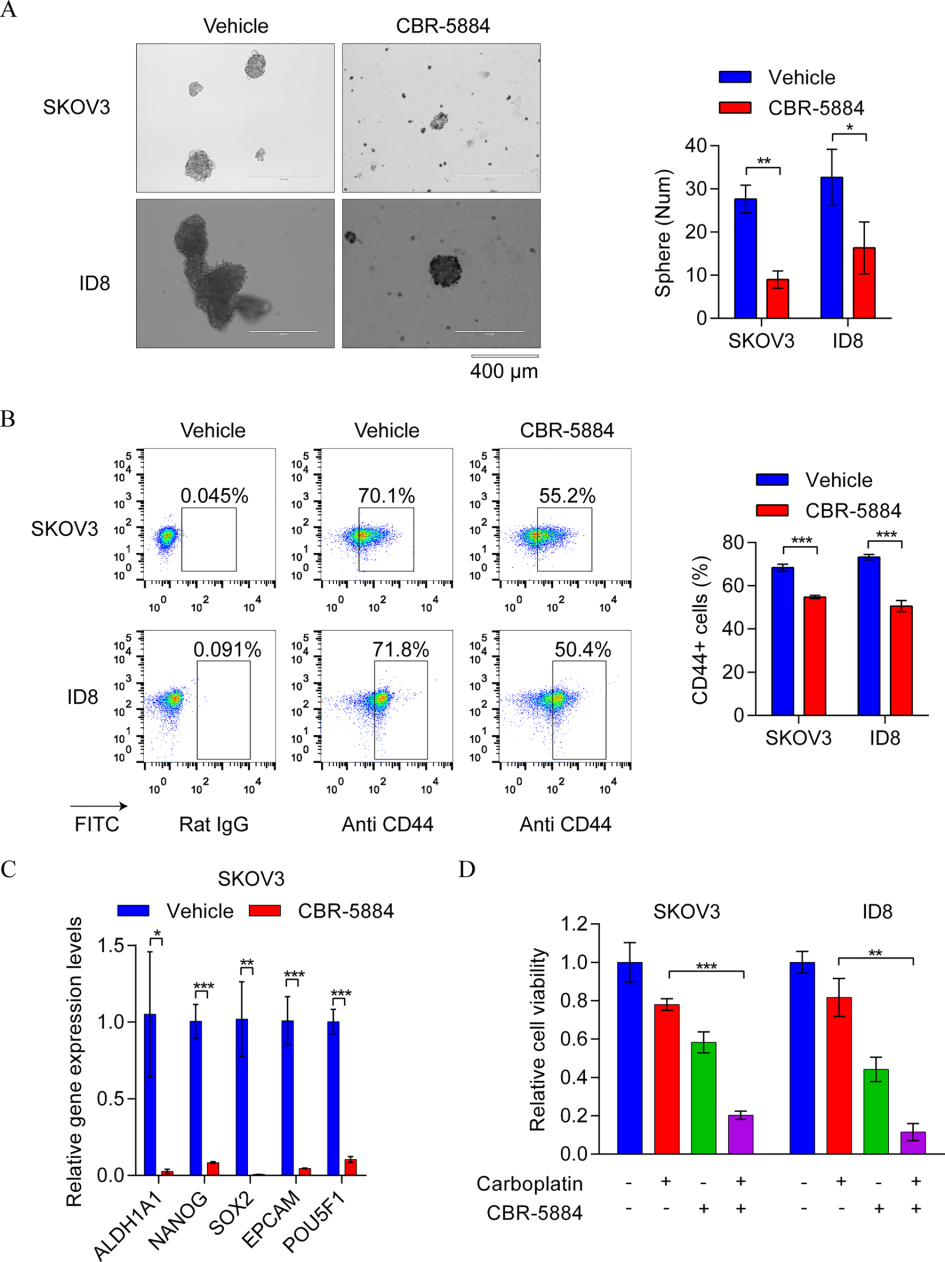
CBR-5884-impaired stemness of EOC cells and enhanced chemotherapy sensitivity. **A** SKOV3 and ID8 cells were treated with CBR-5884 (50 μM) or dimethyl sulfoxide (DMSO; control) for 5 days, and tumorsphere formation ability was accessed by sphere formation assay. Representative images (left) and quantification data (right) are shown. **B** Cell cytometry was performed to detect CD44 expression in SKOV3 and ID8 cells after treatment with 30 μM CBR-5884 for 48 h. Representative images (left) and quantification data (right) are shown. **C** Quantitative polymerase chain reaction assay was conducted to measure the mRNA expression levels of stem-related genes in SKOV3 cells, including *ALDH1A1*, *NANOG*, *SOX2*, *EPCAM*, and *POU5F1* after treatment with 30 μM CBR-5884 for 48 h.** D** SKOV3 and ID8 cells were treated with DMSO, carboplatin (20 μM), CBR-5884 (30 μM), and carboplatin (20 μM) plus CBR-5884 (30 μM) for 72 h. Cell viability was measured using CCK-8 assay

A previous study indicated that increased PHGDH expression contributes to platinum resistance in patients with EOC [20]. Hence, this study sought to determine whether CBR-5884 not only exhibited an antitumor effect in EOC cells but also could enhance chemosensitivity. The stem cell characteristics of tumor cells are closely associated with chemotherapy resistance, and the tumorsphere formation assay is primarily utilized to assess the stemness of tumor cells [30]. To verify this speculation, the tumorsphere formation ability was detected in SKOV3 and ID8 cells treated with either CBR-5884 (50 μM) or DMSO control for 5 days. Remarkably, we observed a significant CBR-5884-mediated suppression of tumorsphere formation in both EOC cell types (Fig. 5A). CD44 serves as a recognized cancer stem cell marker in EOC [31]. Subsequently, we analyzed the protein expression levels of CD44 in EOC cells treated with CBR-5884 (30 μM) or DMSO for 48 h. Flow cytometry results indicated a marked decrease in CD44-positive SKOV3 and ID8 cells upon CBR-5884 treatment (Fig. 5B). Additionally, the expression of stem-related genes, including *ALDH1A1*, *NANOG*, *SOX2*, *EPCAM*, and *POU5F1*, significantly decreased after 48 h of treatment with CBR-5884 (30 μM) in SKOV3 cells (Fig. 5C). Finally, a combination therapy experiment was conducted involving CBP and CBR-5884. Our findings demonstrated that EOC cells treated with CBP plus CBR-5884 exhibited significantly lower cell viability than those receiving CBP alone (Fig. 5D). This underscores the potential of combination treatment strategies in EOC therapy. In conclusion, our findings suggest that CBR-5884 significantly enhances the effect of chemotherapy by suppressing the stem cell characteristics of EOC cells.

### CBR-5884 delayed the proliferation of EOC cells in vivo

**Fig. 6:**
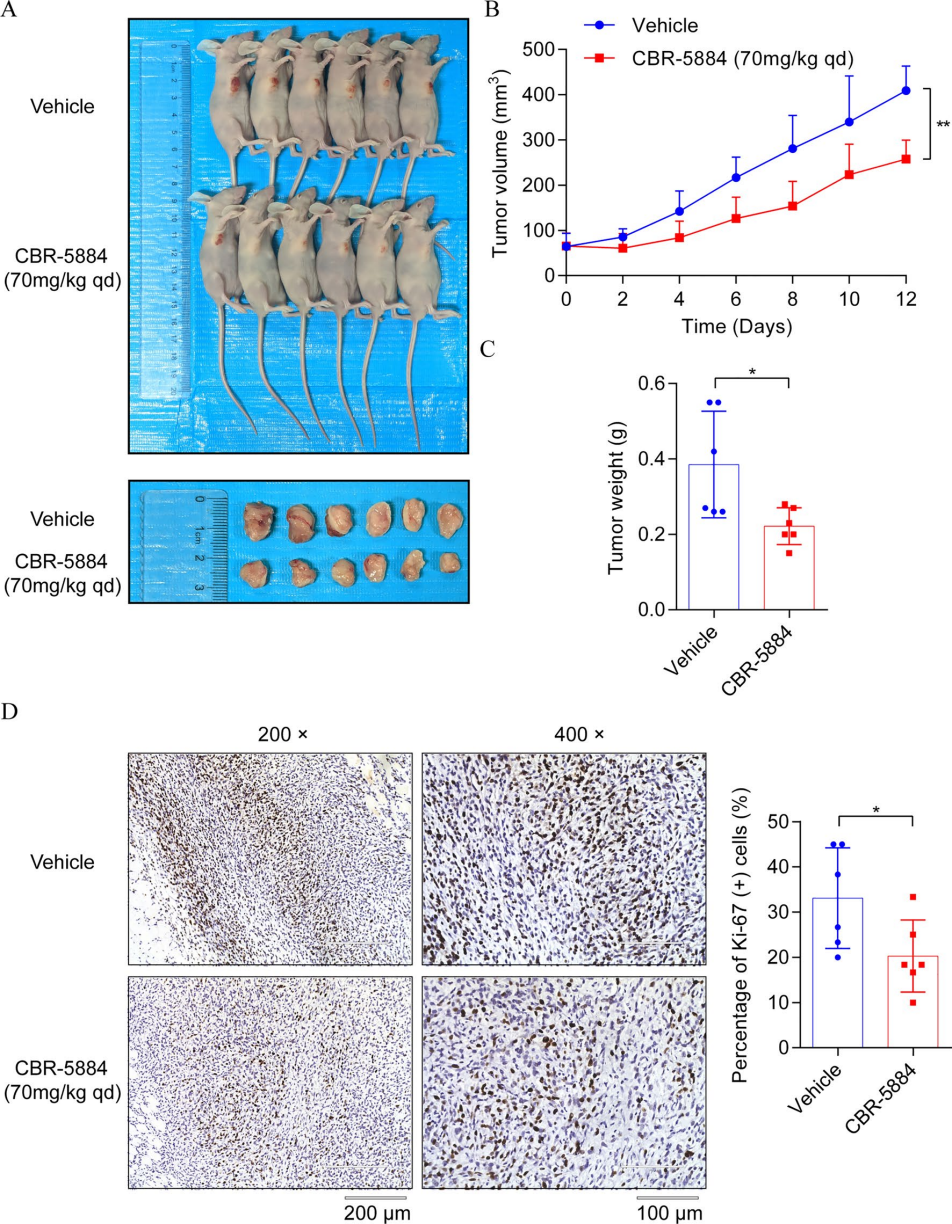
CBR-5884 reduced the tumor growth of EOC cells in vivo. **A** Photographs of autopsy and subcutaneous tumor in mice treated with CBR-5884. **B** Growth curves of subcutaneous tumors in the control and CBR-5884 treatment groups. **C** Tumor weights in the two groups were measured. Each dot represents one sample. **D** Typical images of immunohistochemical detection of Ki-67 expression in subcutaneous tumors in the control and CBR-5884 treatment groups (left). The proportion of Ki-67-positive cells was quantitated (right). Each dot represents one sample

The significant antitumor effect demonstrated by CBR-5884 through cell cycle arrest and apoptosis in vitro prompted us to investigate its inhibitory role in vivo. Subcutaneous injection of ID8 cells into the right armpits of BALB/c nude mice was performed to establish xenograft tumors. In a previous study, CBR-5884 was administered in vivo at a concentration of 50 mg/kg, dissolved in corn oil, and delivered via gavage, and the dosage of CBR-5884 in HEK293T cells was 30 μM [32]. Considering the IC_50_ value of CBR-5884 in ID8 cells is approximately 50 μM, we ultimately decided on an in vivo experimental dosage of 70 mg/kg, also delivered in corn oil using intragastric administration. Once tumors reached an average volume of 65 mm^3^, daily treatment with CBR-5884 at 70 mg/kg commenced, continuing for 12 consecutive days. The results revealed a notable reduction in average tumor volume within the CBR-5884 treated group compared to the control group, despite both groups displaying a gradual increase in tumor size (Fig. 6A, B). Additionally, the average weight of tumors in the CBR-5884 treated group was significantly lower than that in the control group (Fig. 6C). Further analysis using Ki-67 IHC staining confirmed lower proliferation rates in the CBR-5884 treated tumors compared to the control group (Fig. 6D). Moreover, we assessed the expression of PHGDH and ITGB4 in two groups of subcutaneous tumors using IHC. The results indicated that CBR-5884 effectively decreased the expression of PHGDH and ITGB4 in ID8 cells in vivo, consistent with the findings of our in vitro experiments (Fig. S3). This pioneering evaluation of CBR-5884’s anti-tumor effect in vivo prompted an assessment of its safety. Throughout the experiment, we monitored and compared the weight of mice in both groups. The results indicated a similar weight trend between the two groups (Fig. S4A), with no reduction observed in mouse weight following CBR-5884 treatment (Fig. S4B), suggesting that the in vivo application of CBR-5884 did not affect the weight of mice. Additionally, H&E staining revealed no evident changes in the livers, spleens, or kidneys between the two groups (Fig. S4C).

### CBR-5884 suppresses EOC tumorigenesis in PDO models

**Fig. 7:**
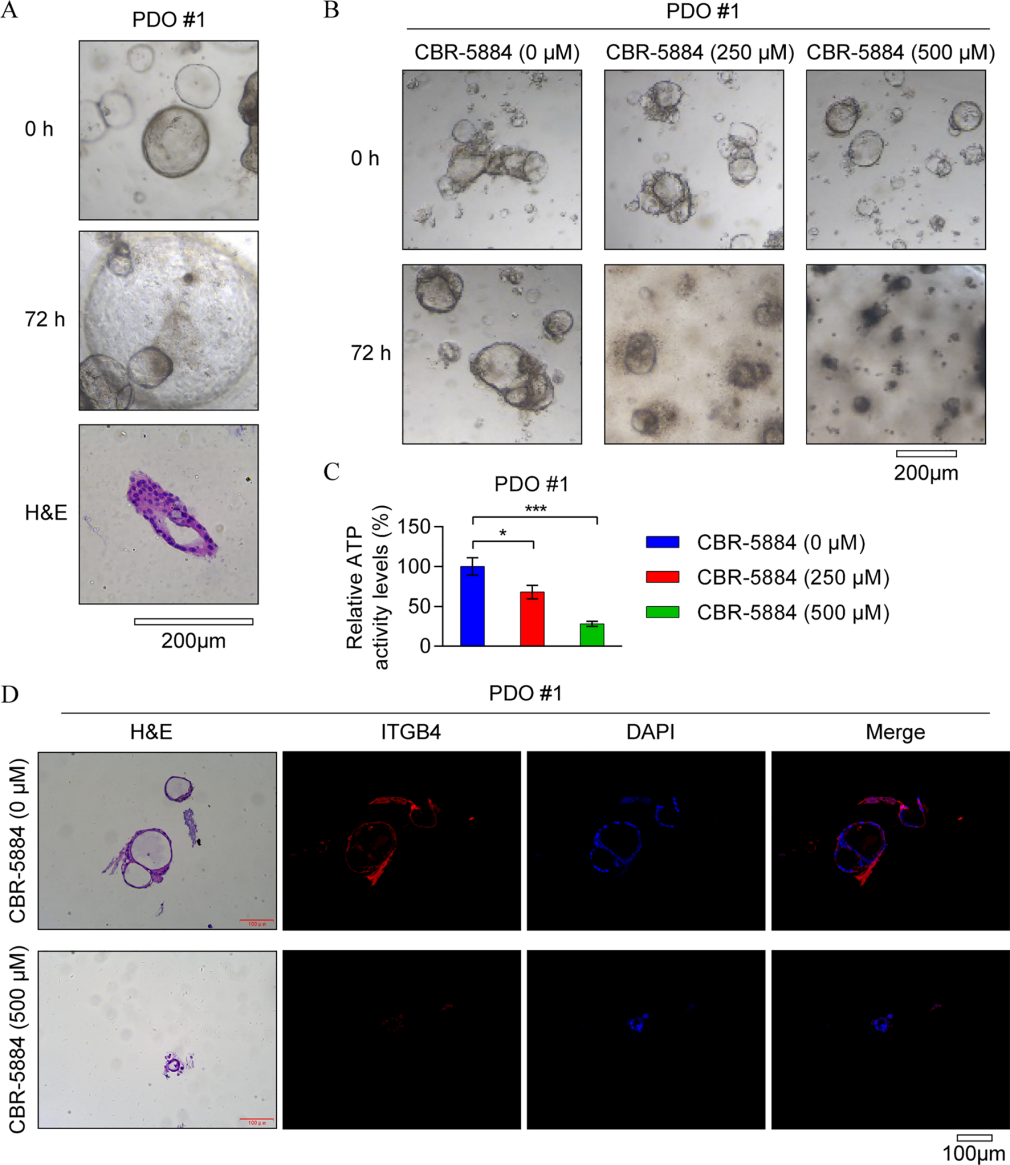
CBR-5884 impaired tumorigenesis in EOC PDO models. **A** Representative images of PDO models derived from tumor tissue of patients with EOC. **B**, **C** EOC PDO models were treated with different concentrations of CBR-5884 (0 μM, 250 μM, and 500 μM) for 72 h, and ATP detection assay was performed to detect the inhibitory effect of CBR-5884 treatment on cell proliferation. Representative images (**B**) and quantification data (**C**) are shown. **D** Immunofluorescence assay was performed to detect ITGB4 protein levels in PDO models treated with CBR-5884 (0 μM and 500 μM) for 72 h

PDO models have emerged as key preclinical systems for assessing drug responses and efficacy. Initially generated from tumor tissues obtained from a single patient with EOC, the number and volume of PDOs progressively increased over time, demonstrating their utility in evaluating the preclinical efficacy of CBR-5884 (Fig. 7A). Subsequent treatment of these PDO cells with 250 μM or 500 μM CBR-5884 for 72 h revealed a marked dose-dependent inhibition of viability in EOC PDO cells (Fig. 7B, C). Furthermore, through immunofluorescence detection of intracellular ITGB4 expression levels, we found that treatment with high concentrations of CBR-5884 significantly downregulated ITGB4 expression in those PDO cells (Fig. 7D), which is consistent with the results in EOC cell lines. Collectively, these results provide preliminary evidence that CBR-5884 exerts a significant anti-tumor effect with favorable safety profiles.

## Discussion

Recent studies emphasize that metabolic reprogramming sustains tumor cell development [33–35]. Abnormal activation of metabolic enzymes mediates metabolic reprogramming, facilitating malignant progression in tumors [36]. PHGDH, a pivotal enzyme in the serine biosynthesis pathway, is highly expressed in OC. However, abnormal PHGDH activation promotes tumor progression [37]. PHGDH knocking down inhibits the metastatic ability and stemness of EOC cells [20], suggesting that targeting PHGDH or overactive serine metabolism might be a potential strategy for treating OC. Our study confirms, for the first time, that the selective PHGDH inhibitor CBR-5884 significantly inhibits serine biosynthesis in EOC cells at the transcriptional level. Additionally, due to the suppression of SSP, cellular steroid hormone biosynthesis, nucleotide biosynthesis, and one-carbon unit metabolism were partially repressed by CBR-5884 treatment, indicating its targeted inhibition of serine metabolic reprogramming in EOC cells. Analysis of sequencing data also revealed the upregulation of gene sets related to the tumor necrosis factor (TNFα) signaling pathway and inflammatory response due to CBR-5884 treatment. Studies suggest that manipulating serine metabolism can serve as a therapeutic strategy against viral infections [32]. Further investigation is necessary to ascertain whether CBR-5884 induces inflammation and its effect on the immune system or immunotherapy.

CBR-5884, functioning as a noncompetitive inhibitor, demonstrated time-dependent inhibition, disrupting the oligomerization state of PHGDH. It effectively impeded the proliferation of breast cancer cells with a high serine propensity [21]. This study also unveiled its inhibitory effect on the proliferation of EOC cells expressing elevated levels of PHGDH. Similar to many targeted drugs, CBR-5884 displayed significant antitumor efficacy against EOC cells in vitro, manifesting through cell cycle blockade, apoptosis induction, and inhibition of cell invasion and metastasis. However, its efficacy in inhibiting tumor growth in nude mice was found to be unsatisfactory. During the drug preparation process, we observed that CBR-5884 readily precipitated in corn oil. We hypothesize that the gavage administration using corn oil as a vehicle may not facilitate the complete absorption of the experimental dosage of CBR-5884 by mice, potentially contributing to its weakened in vivo efficacy. Nonetheless, our findings revealed minimal damage to vital organs in mice and overall good health, indicating the safety of CBR-5884 treatment in vivo. Consequently, we intend to modify CBR-5884 in subsequent investigations to enhance its solubility or explore alternative routes of administration, thereby further elucidating the in vivo efficacy of CBR-5884 or its derivatives.

Integrins, composed of two noncovalently associated transmembrane glycoprotein subunits, alpha and beta, belong to cell surface transmembrane glycoprotein receptors [38]. ITGB4, a member of the integrin family, is reported as a poor prognostic indicator for patients with EOC [39]. In our research, following CBR-5884 treatment, we observed significant downregulation of mRNA expression levels of integrin family members, notably ITGB4, in SKOV3 cells. This downregulation might be associated with the inhibition of cellular nucleotide synthesis. However, the precise molecular mechanism remains unclear and necessitates further investigation. Understanding how CBR-5884 inhibits integrins would add a new theoretical dimension to the role of serine metabolic reprogramming in EOC. In addition to the integrin family members, we have observed that CBR-5884 reduced the transcription levels of many genes. Further experiments are needed to elucidate whether the downregulation of these genes is due to the decreased nucleotide availability or to the downregulation of integrin family members by CBR-5884. Meanwhile, we also found that the expression of some genes is upregulated after CBR-5884 treatment. We speculate that this may be a feedback upregulation resulting from decreased nucleotide availability in cells following inhibition of SSP. Therefore, it is necessary to investigate in depth the specific molecular mechanisms by which CBR-5884 affects gene transcription levels. Currently, it is understood that CBR-5884 acts as an inhibitor of PHGDH, and our findings validated its pronounced inhibitory impact on serine synthesis. However, further investigation is required to ascertain whether CBR-5884 targets other rate-limiting enzymes of the SSP.

Chemotherapy resistance remains a critical factor in therapeutic failures among patients with EOC. Amino acid metabolism reprogramming sustains stem cell-like characteristics and drug resistance in EOC cells [40, 41]. Notably, our findings indicate that CBR-5884 reduced tumorsphere formation ability and CD44 expression levels, suggesting a decrease in the stemness of OC cells. Furthermore, combining CBR-5884 with CBP, a first-line clinical drug against EOC, revealed a significantly synergistic effect. These outcomes suggest that CBR-5884 could be an alternative therapy against chemotherapy resistance in EOC. In summary, our study demonstrates that CBR-5884 effectively targets active serine metabolism in EOC cells for the first time, exhibiting remarkable antitumor effects in EOC cells with high PHGDH levels. Additionally, CBR-5884-mediated SSP inhibition enhances chemosensitivity, thereby presenting CBR-5884 as a potential therapeutic agent for EOC. Moreover, developing small molecule inhibitors targeting serine synthesis based on CBR-5884 could greatly aid clinical treatment of EOC.

## Conclusions

The selective PHGDH inhibitor, CBR-5884, inhibits serine biosynthesis and related metabolic processes in EOC cells, effectively exerting an antitumor effect by inducing cell cycle arrest and apoptosis. Moreover, CBR-5884 suppresses the migration and invasion of EOC cells by targeting the ITGB4/ERK/EMT signaling axis, enhancing chemotherapy sensitivity. Our in vivo and PDO model experiments confirm the safety and potential clinical value of CBR-5884.

### Supplementary Information

Below is the link to the electronic supplementary material.

Supplementary file 1 (DOCX 6368 KB)

Supplementary file 2 (PDF 16331 KB)

Supplementary file 3 (XLS 36826 KB)

## Data Availability

The datasets supporting the conclusions of this article are available on the GEPIA website, http://gepia.cancer-pku.cn/; the Kaplan–Meier Plotter website, https://kmplot.com/analysis/; the UCSC Xena TCGA hub repository, https://xenabrowser.net/; and the GEO repository (code GSE14407). Further information and requests for reagents and resources should be directed to and will be made available by the corresponding authors upon reasonable request.

## Abbreviations

EOC: Epithelial ovarian cancer
SSP: Serine synthesis pathway
PHGDH: Phosphoglycerate dehydrogenase
ITGB4: Integrin subunit beta 4
EMT: Epithelial–mesenchymal transition
ERK: Extracellular signal-regulated kinase
DMEM: Dulbecco’s modified Eagle’s medium
FBS: Fetal bovine serum
GSEA: Gene set enrichment analysis
FACS: Fluorescence activated cell sorter
TCGA: The Cancer Genome Atlas
CCK-8: Cell counting kit-8
CBP: Carboplatin
