# Supplementary material for: Preclinical efficacy of CBR-5884 against epithelial ovarian cancer cells by targeting the serine synthesis pathway
Source: Discov Oncol. 2024 May 11;15:154. doi: 10.1007/s12672-024-01013-0 (PMC11088592; doi:10.1007/s12672-024-01013-0)
Supplement: Supplementary file 1 — (DOCX 6368 KB) [file 12672_2024_1013_MOESM1_ESM.docx]

***Supplementary Information***

**Preclinical efficacy of CBR-5884 against epithelial ovarian cancer cells by targeting the serine synthesis pathway**

**Supplementary Figures**

**
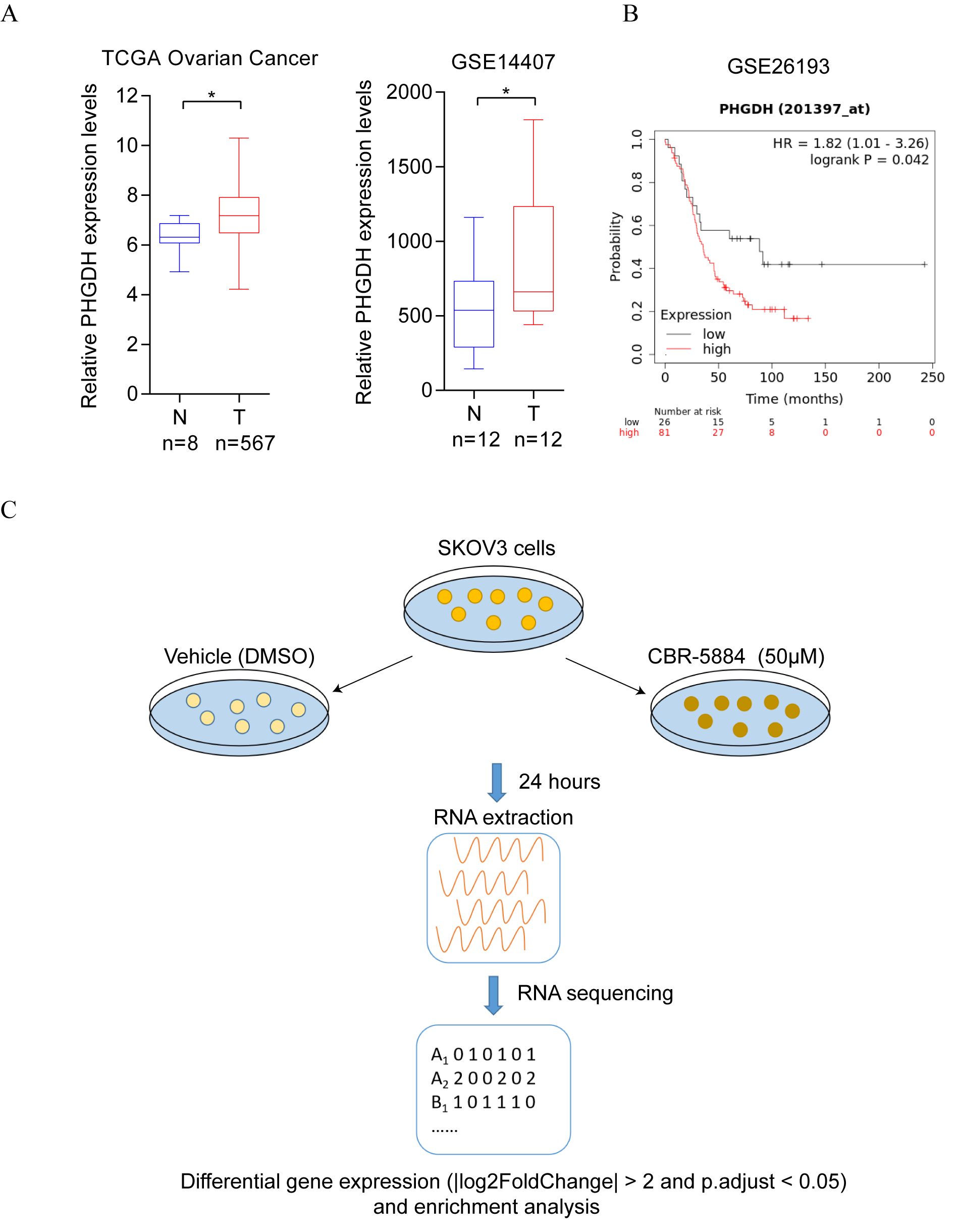
**

**Fig. S1. Role of phosphoglycerate dehydrogenase (PHGDH) in ovarian cancer cells.**

1. PHGDH expression level in patients with ovarian cancer in The Cancer Genome Atlas (TCGA) database and GSE14407.
2. Overall survival was analyzed with the log-rank test for PHGDH high/low groups of patients with ovarian cancer obtained from GSE26193.
3. Flowchart of transcriptome sequencing.

**
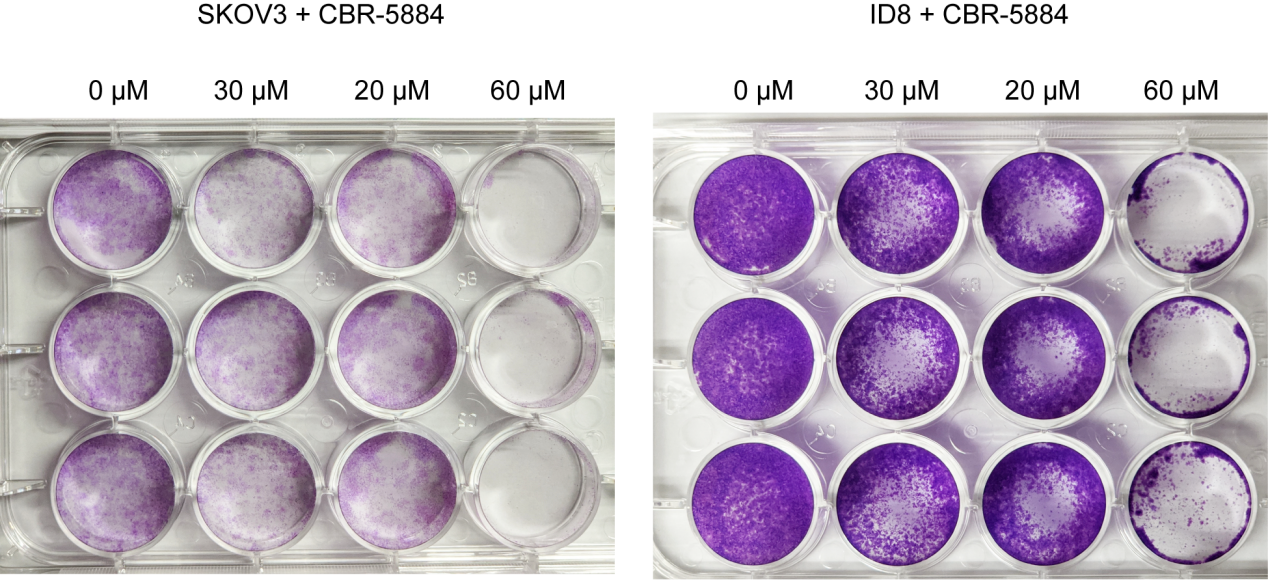
**

**Fig. S2. The whole plates of Figure 2G.**


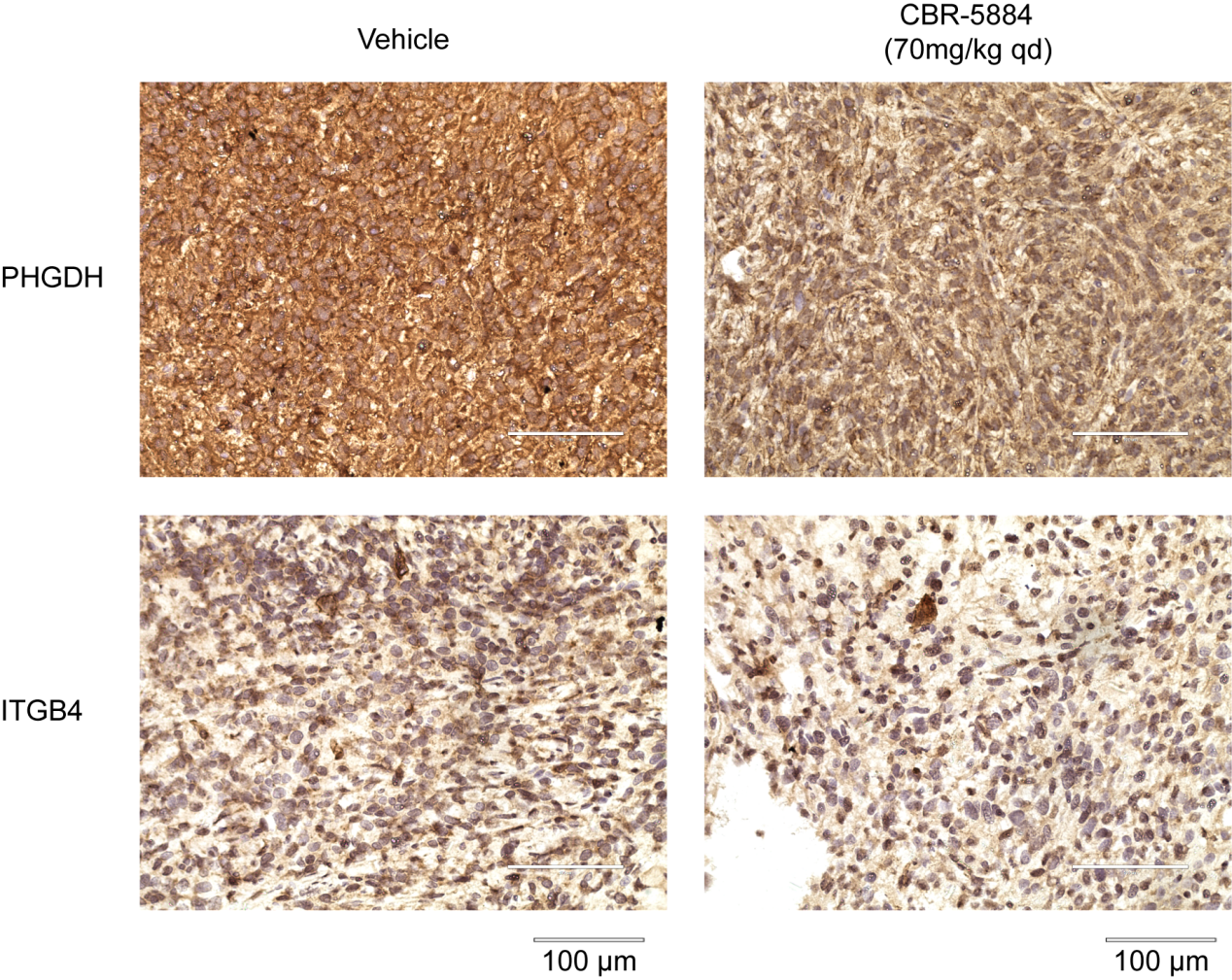


**Fig.S3. Typical images of immunohistochemical detection of PHGDH (*Upper*) and ITGB4 (*bottom*) expression in subcutaneous tumors in the control and CBR-5884 treatment groups.**

**
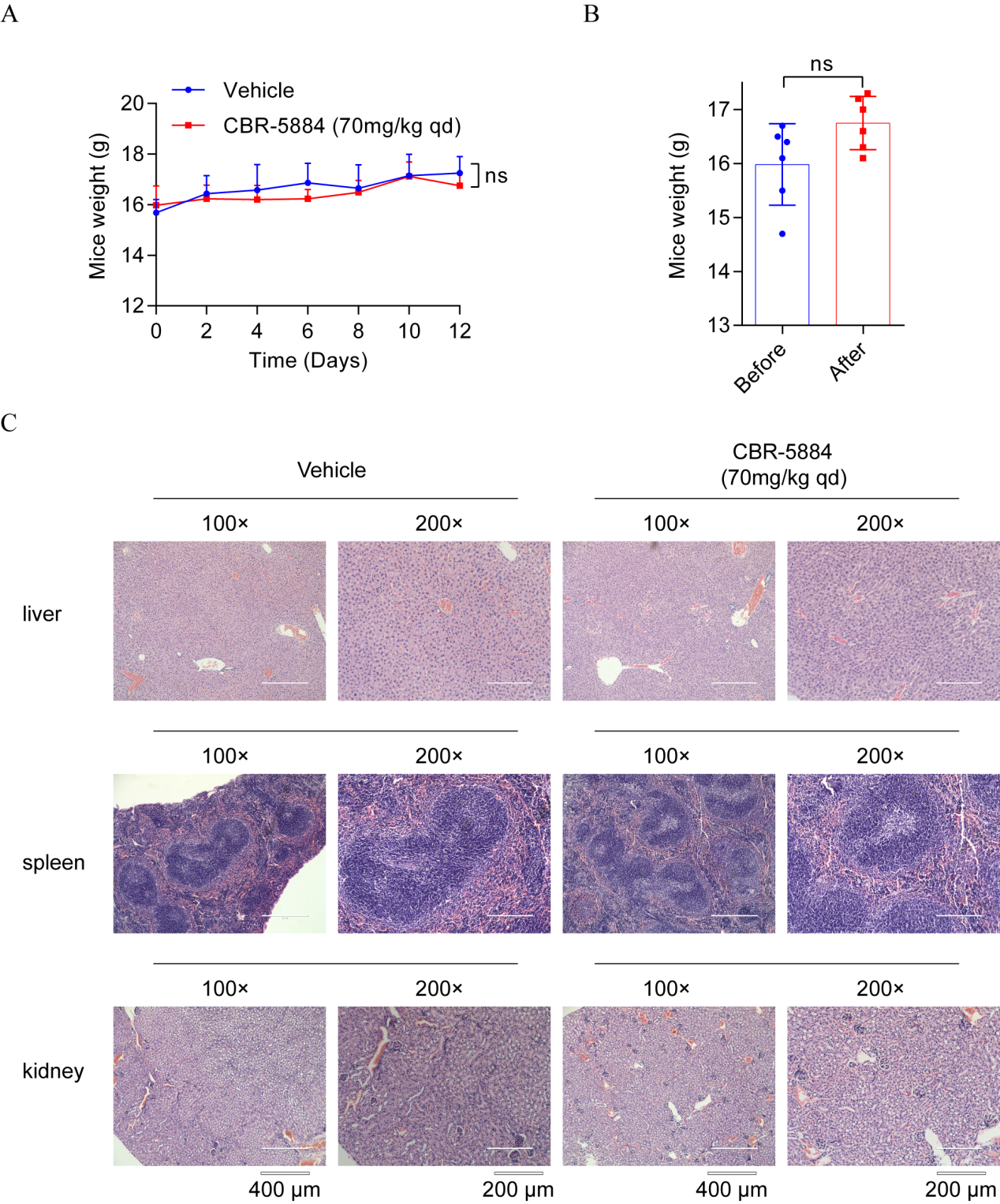
**

**Fig. S4. Assessment of *in vivo* safety of CBR-5884.**

1. Comparison of body weight of nude mice between the control and CBR-5884 treatment groups.
2. Comparison of body weight of nude mice before and after CBR-5884 treatment; each dot represents one sample.
3. Hematoxylin-eosin staining was performed to assess changes in the liver, spleen, and kidney of mice before and after CBR-5884 treatment.

**Supplementary Table**

**Table S1. Patient information**

| **Patient number** | **Age** | **Histologic type** | **Sstage** | **With/without chemotherapy** | **Purpose** |
| --- | --- | --- | --- | --- | --- |
| **1** | **54** | **Serous tumor** | **Ⅰc** | **No chemotherapy** | **PDO** |
