# Supplementary material for: Preclinical efficacy of CBR-5884 against epithelial ovarian cancer cells by targeting the serine synthesis pathway
Source: Discov Oncol. 2024 May 11;15:154. doi: 10.1007/s12672-024-01013-0 (PMC11088592; doi:10.1007/s12672-024-01013-0)

Full scans of western blots

Preclinical efficacy of CBR-5884 against epithelial ovarian cancer cells by targeting the serine synthesis pathway

Gong *et al.*

The raw data of western blots in this study are original, uncropped and unedited.

The representative blots which displayed in the corresponding Figure were marked in the red boxes.

Figure. 2B

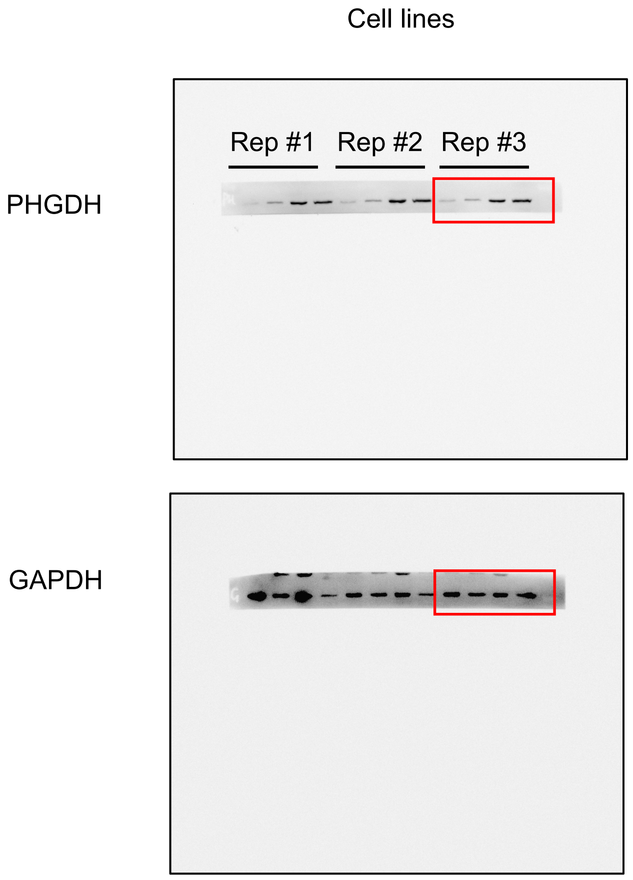

Figure. 3C

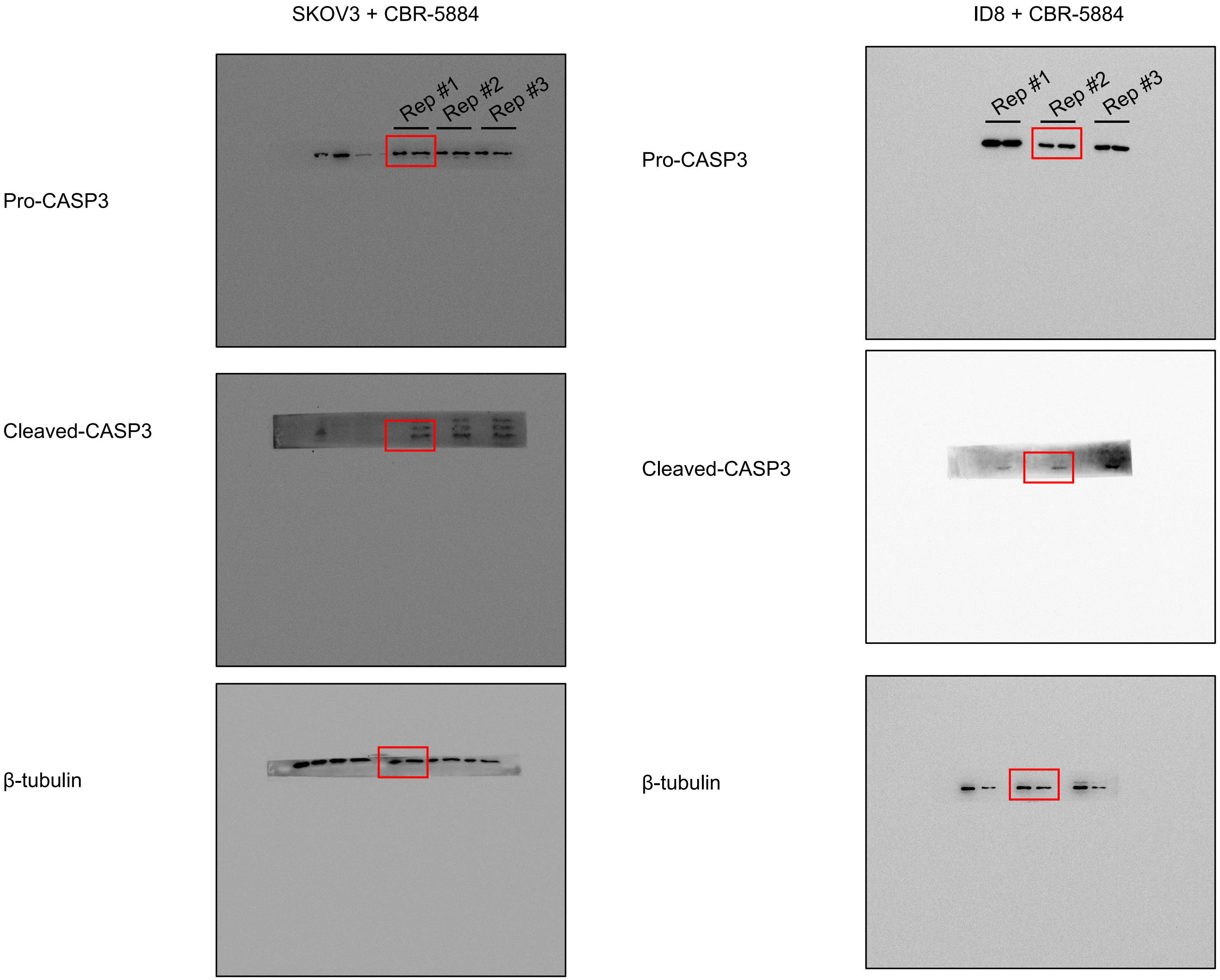

Figure. 4D

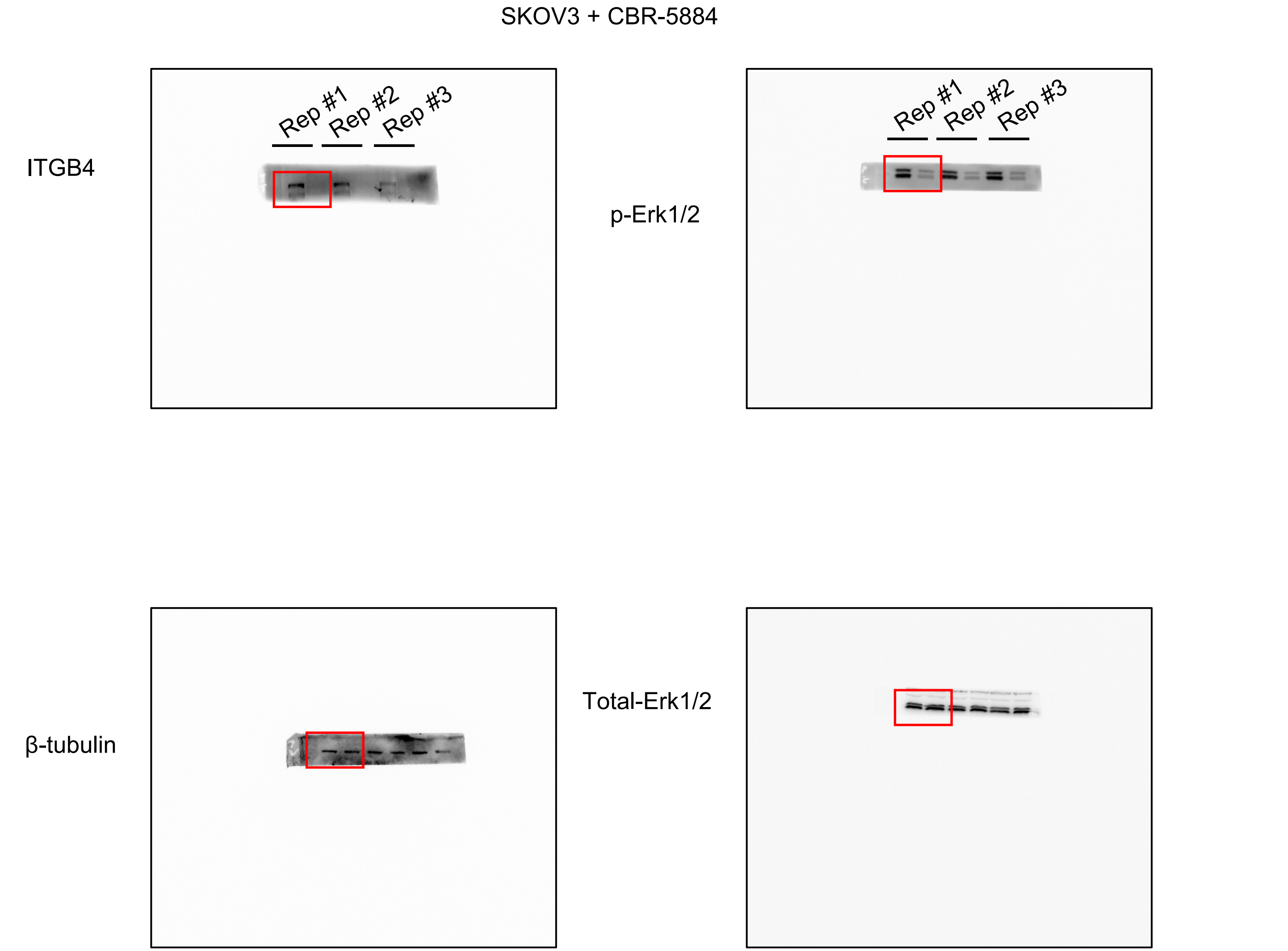
SKOV3 + CBR-5884
ITGB4
Rep #1
Rep #2
Rep #3
p-Erk1/2
Rep #1
Rep #2
Rep #3
β-tubulin
Total-Erk1/2

Figure. 4E

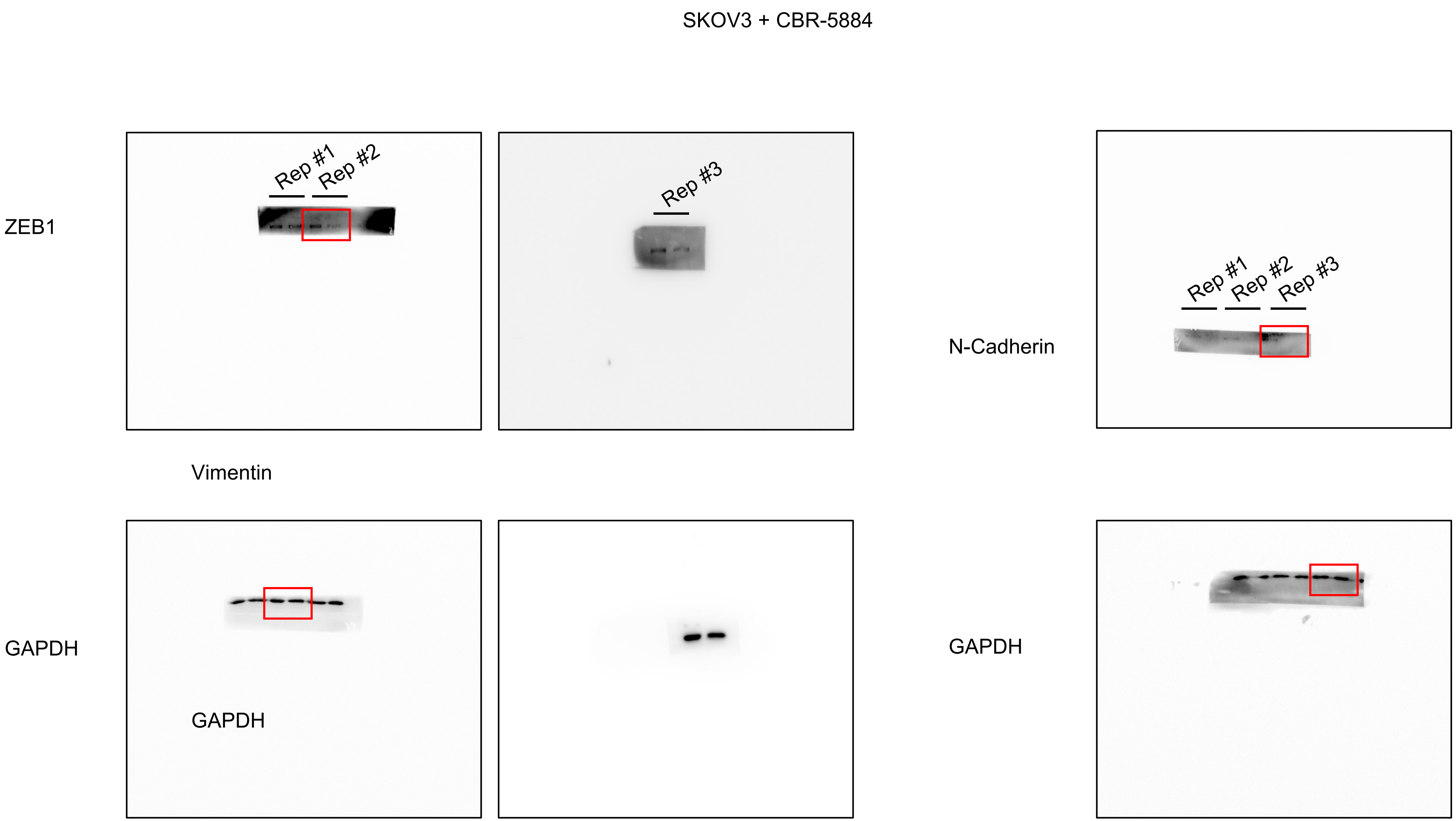
SKOV3 + CBR-5884
ZEB1
Rep #1
Rep #2
Rep #3
N-Cadherin
Rep #1
Rep #2
Rep #3
Vimentin
GAPDH
GAPDH
GAPDH

Figure. 4E

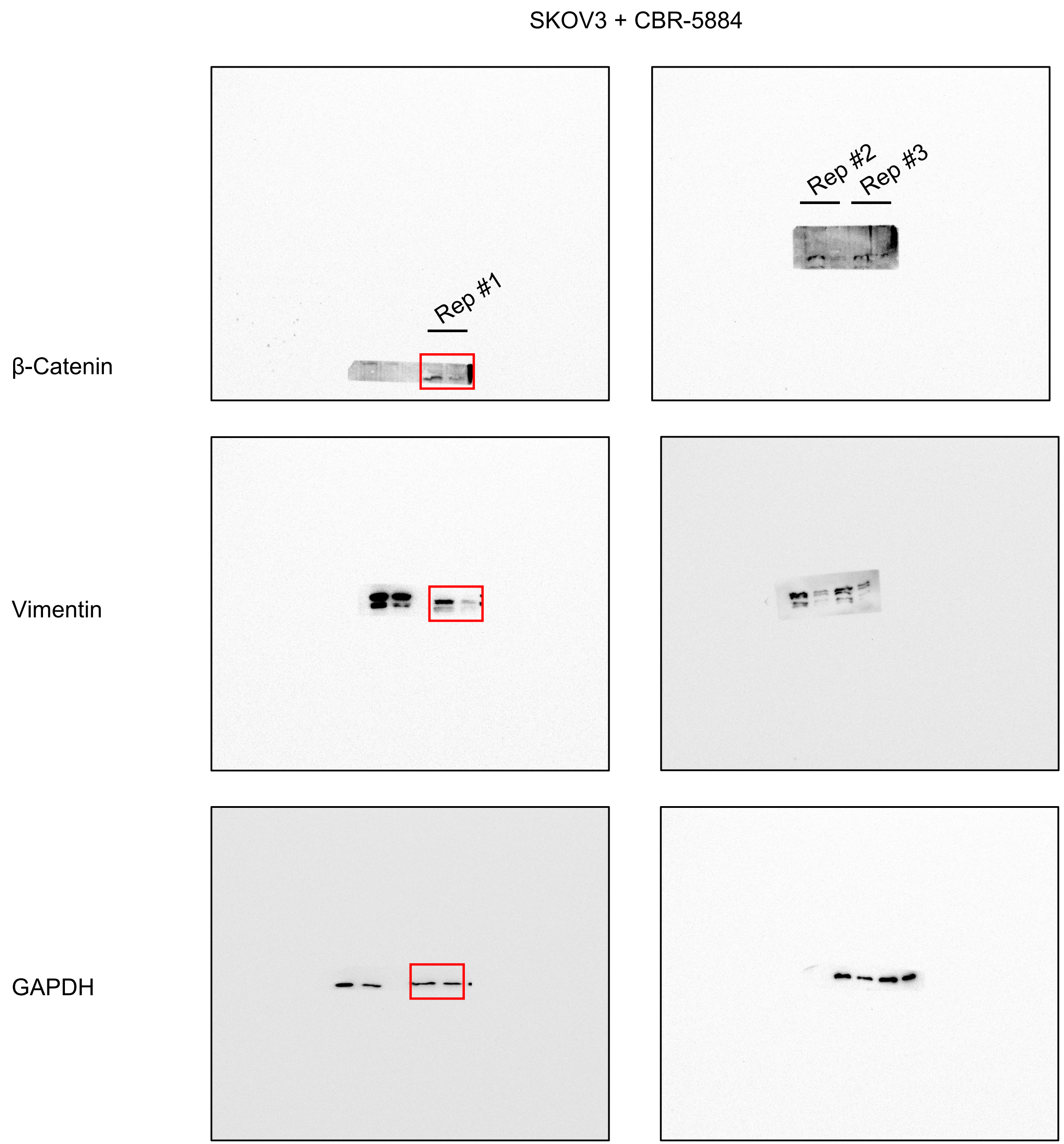

Figure. 4E

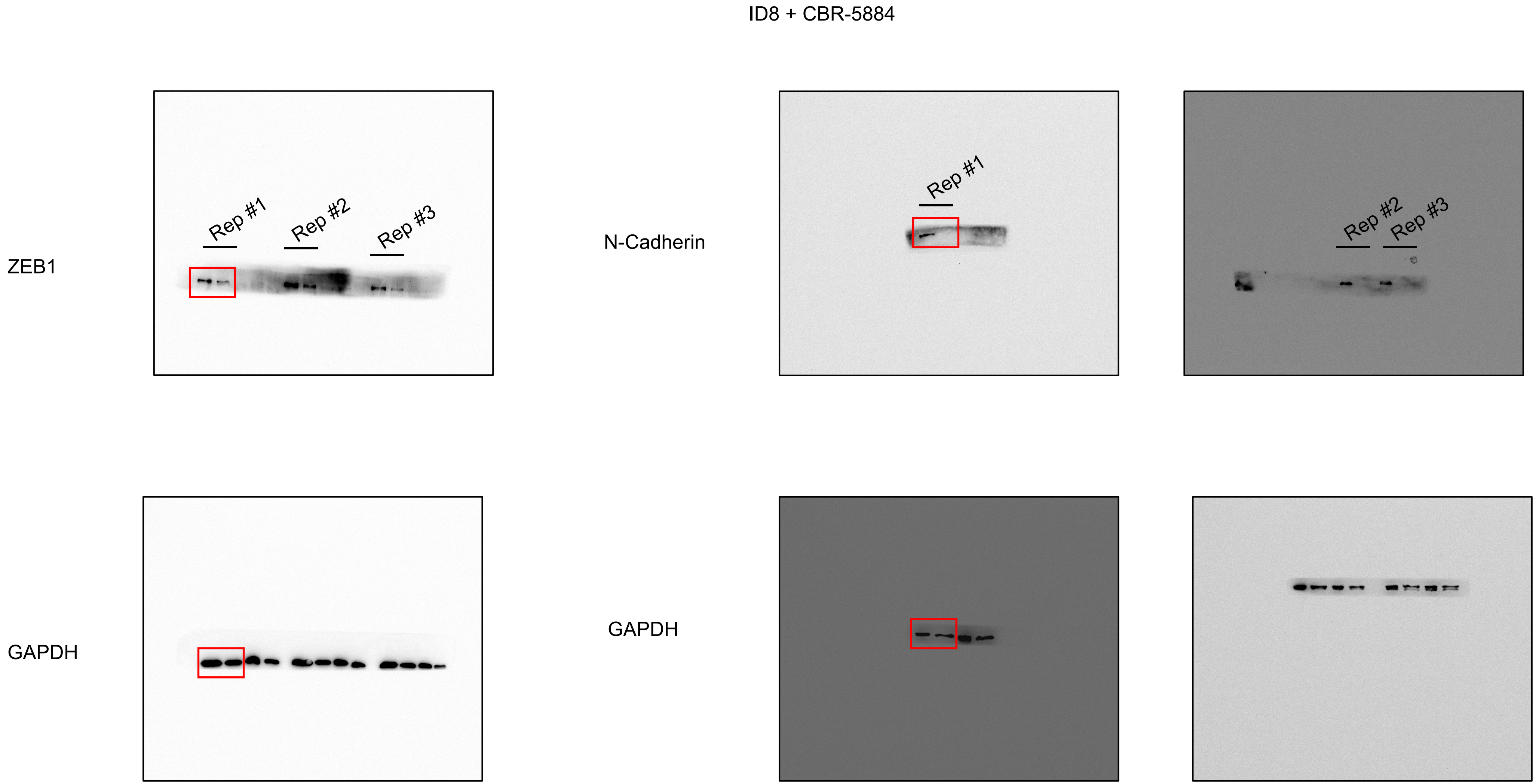
ID8 + CBR-5884
ZEB1
Rep #1
Rep #2
Rep #3
N-Cadherin
Rep #1
Rep #2
Rep #3
GAPDH
GAPDH

Figure. 4E

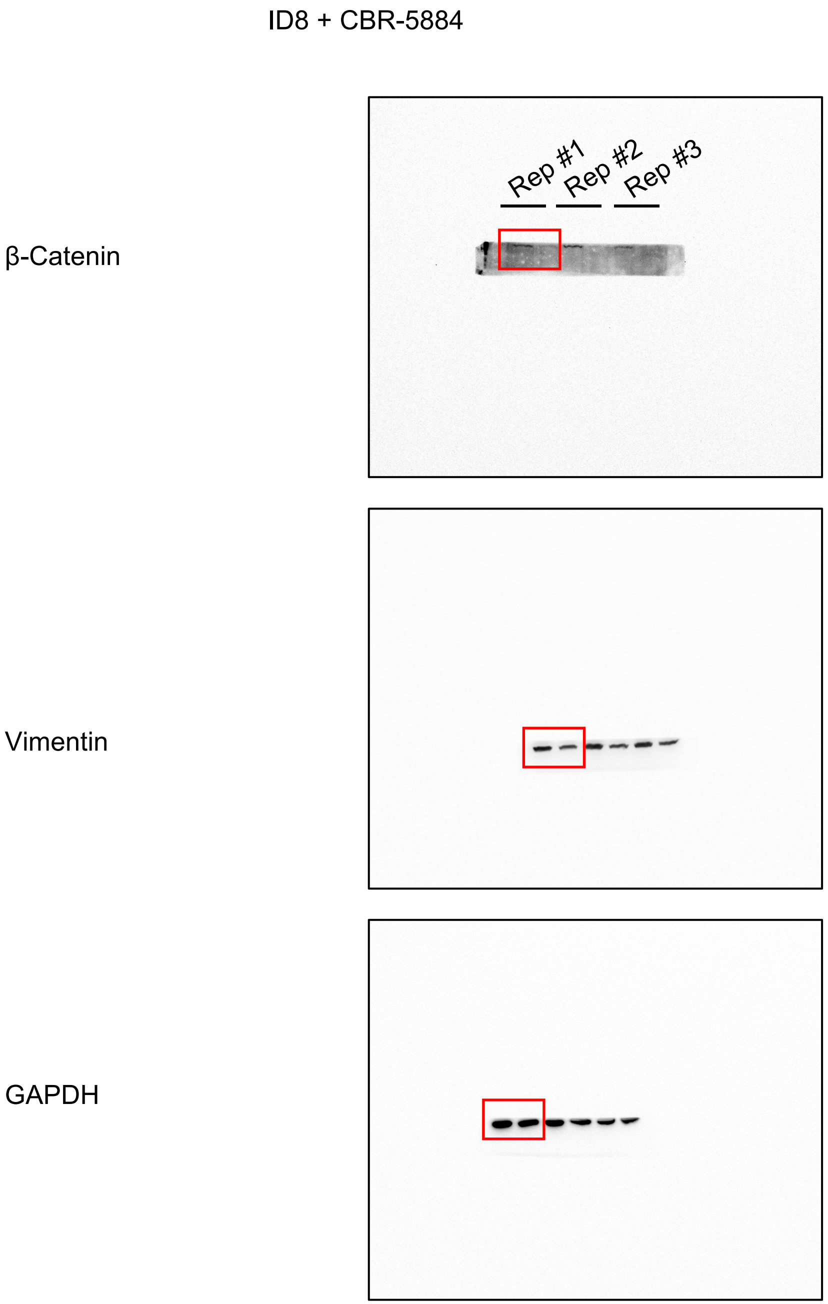

Supplement: Supplementary file 2 — (PDF 16331 KB) [file 12672_2024_1013_MOESM2_ESM.pdf]
